# Robustness of DNA Repair through Collective Rate Control

**DOI:** 10.1371/journal.pcbi.1003438

**Published:** 2014-01-30

**Authors:** Paul Verbruggen, Tim Heinemann, Erik Manders, Gesa von Bornstaedt, Roel van Driel, Thomas Höfer

**Affiliations:** 1 Division of Theoretical Systems Biology, German Cancer Research Center (DKFZ), Heidelberg, Germany; 2 Swammerdam Institute for Life Sciences, University of Amsterdam, Amsterdam, The Netherlands; 3 BioQuant Center, Heidelberg, Germany; ETH Zurich, Switzerland

## Abstract

DNA repair and other chromatin-associated processes are carried out by enzymatic macromolecular complexes that assemble at specific sites on the chromatin fiber. How the rate of these molecular machineries is regulated by their constituent parts is poorly understood. Here we quantify nucleotide-excision DNA repair in mammalian cells and find that, despite the pathways' molecular complexity, repair effectively obeys slow first-order kinetics. Theoretical analysis and data-based modeling indicate that these kinetics are not due to a singular rate-limiting step. Rather, first-order kinetics emerge from the interplay of rapidly and reversibly assembling repair proteins, stochastically distributing DNA lesion repair over a broad time period. Based on this mechanism, the model predicts that the repair proteins collectively control the repair rate. Exploiting natural cell-to-cell variability, we corroborate this prediction for the lesion-recognition factor XPC and the downstream factor XPA. Our findings provide a rationale for the emergence of slow time scales in chromatin-associated processes from fast molecular steps and suggest that collective rate control might be a widespread mode of robust regulation in DNA repair and transcription.

## Author Summary

The nucleotide-excision repair pathway removes mutagen-inflicted DNA lesions from the genome. Repair proteins recognize DNA lesions and form multi-protein complexes that catalyze the excision of the lesion and the re-synthesis of the excised part. Imaging the dynamics of fluorescently labeled repair proteins in living human cells has revealed that all factors continuously and rapidly exchange at repair sites. We asked how this dynamic mode of protein-complex assembly shapes the repair process. Measuring repair DNA synthesis in intact cells, we obtained a surprisingly simple result. Over the entire process, the rate is proportional to the amount of DNA lesions, where the proportionality factor is a single ‘slow’ rate constant. Such kinetic behavior is often regarded as evidence for a rate-limiting step, but we show here that it is an emergent property of the dynamic interplay of many repair proteins. As a consequence, the rate of DNA repair is a systems property that is controlled collectively by the expression levels of all repair factors. Given that transcription in living cells has similar dynamic features – rapidly exchanging components of the transcription machinery and slow bursts of mRNA synthesis – collective rate control might be a general property of chromatin-associated molecular machines.

## Introduction

Chromatin-associated processes, including transcription, replication, DNA repair and epigenetic control, are executed by macromolecular complexes that assemble at specific sites on the chromatin fiber. The stepwise, cooperative ‘recruitment’ of components into stable complexes has, until recently, been the prevailing model for the assembly of these macromolecular machineries. In contrast to this notion, imaging of transcription and DNA repair in living cells has shown that the constituents of the respective complexes dissociate and rebind at the seconds-to-minutes time scale at specific sites of transcription initiation and DNA damage [1], [2] and that RNA polymerase clustering in transcription factories is dynamic on a similar time scale [3]. Similar findings have been made for replication, with the exception of stably bound Mcm proteins which mark licensed replication origins [4], [5]. Based on these data, an alternative model for the assembly of transcription-initiation and DNA repair complexes has been proposed: functional complexes form from randomly diffusing and rapidly exchanging components [6]–[8]. The kinetic and functional implications of this highly dynamic mode of macromolecular complex formation on DNA remain poorly understood.

Combining experiments on living cells with mathematical modeling, several studies have begun to unravel the relation between assembly dynamics and functionality of chromatin-associated machineries. Gorski et al. [9] demonstrated that moderate dwell-time changes of RNA polymerase components at transcription initiation regulate transcription rate. Voss et al. [10] showed that a rapidly exchanging transcription factor facilitates the subsequent binding of a second transcription factor by enzymatically increasing chromatin accessibility. In this “assisted-loading” mechanism, the first transcription factor enhances the binding of the second one via the modification of the chromatin template, thus obviating the need for simultaneous and cooperative binding. A comprehensive live-cell imaging study of nucleotide-excision repair (NER) in mammalian cells predicted that the rapid exchange of NER factors allows for high specificity of the pathway through kinetic proofreading [8].

**Figure 1:**
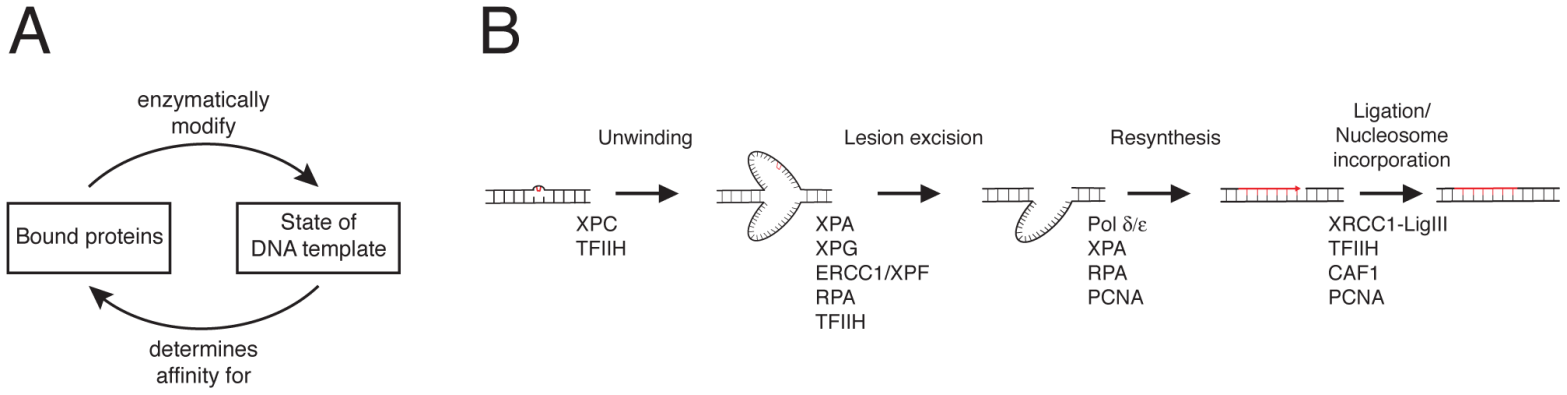
Chromatin-associated processes. (**A**) Principle of the sequential organization of chromatin-associated processes through cycles of protein recruitment, enzymatic modification and subsequent reorganization of the protein machinery. (**B**) Nucleotide excision repair in mammalian cells. Shown are the sequence of repair steps and, for each step, the proteins that need to assemble on the DNA to catalyse it.

A common theme linking these studies is the functional coupling between protein binding and modifications of chromatin by the bound proteins (including both DNA and protein modifications). This interplay suggests a cyclic principle (Fig. 1A): Appropriately assembled multi-protein complexes drive enzymatic reactions that modify the chromatin template, in turn changing protein affinities for the assembly of the next enzymatic complex. Multiple ‘recruitment-reaction’ cycles of this kind can integrate random and transient diffusion-dependent protein binding steps into an ordered sequence of regulatory events at a specific chromatin site [11]. Based on *in vivo* experiments, a detailed mathematical model of this type has been developed for NER in mammalian cells [8]. NER efficiently recognizes and removes a wide variety of DNA lesions from the damaged DNA strand and resynthesizes the excised part by using the intact strand as a template [2]. Live-cell imaging has shown that all core proteins for global-genome NER exchange rapidly at damaged DNA sites in the cell nucleus as repair takes place [8], [12]–[15]. The resulting model describes the NER pathway as the sequential conversion of DNA intermediates – damage recognition followed by DNA unwinding, lesion excision, re-synthesis and ligation of the excised strand – with each conversion step catalysed by a specific multi-protein complex that assembles reversibly at the site of repair when needed (Fig. 1B).

The NER model by Luijsterburg et al. [8] was able to integrate a large data set for all core NER proteins because a standard protocol was employed for each of them, consisting of inflicting DNA lesions locally in the cell nucleus by a pulse of UV light and subsequent monitoring of the repair factor dynamics by live-cell imaging [13], [16]. While the model presents a quantitative description of the *in vivo* data, the data themselves were not sufficient to uniquely identify all kinetic parameters (on-rate and off-rate constants as well as enzymatic rate constants). Therefore quantitative predictions with the model on the functioning of NER in intact cells were limited. This limitation of predictive power is due the fact that the model is based on measured assembly rates and dwell times of the repair factors whereas the repair of the DNA lesions has not been measured with comparable time resolution (similar limitations may apply to imaging-based models of transcription dynamics [9], [17], [18]).

Here we quantify the relation between the protein dynamics measured previously and the resulting DNA repair by measuring DNA repair synthesis in a time-resolved manner in individual living cells. We find that lesion repair by NER is effectively a slow first-order reaction and show this to be an emergent property of the fast dynamics of the repair proteins. The new data allow the development of an identifiable and hence predictive NER model. A conspicuous quantitative prediction of the model is that NER does not have a singular rate-limiting step but, instead, that the concentrations of all core repair factors have small or moderate effect on – and thus collectively control – the repair rate. Harnessing natural cell-to-cell variability of protein expression levels, we provide experimental support for this prediction. Our study introduces a quantitative framework for dissecting the dynamics of macromolecular machines that act on the genome.

## Results

### Kinetics of DNA repair synthesis in intact cells

**Figure 2:**
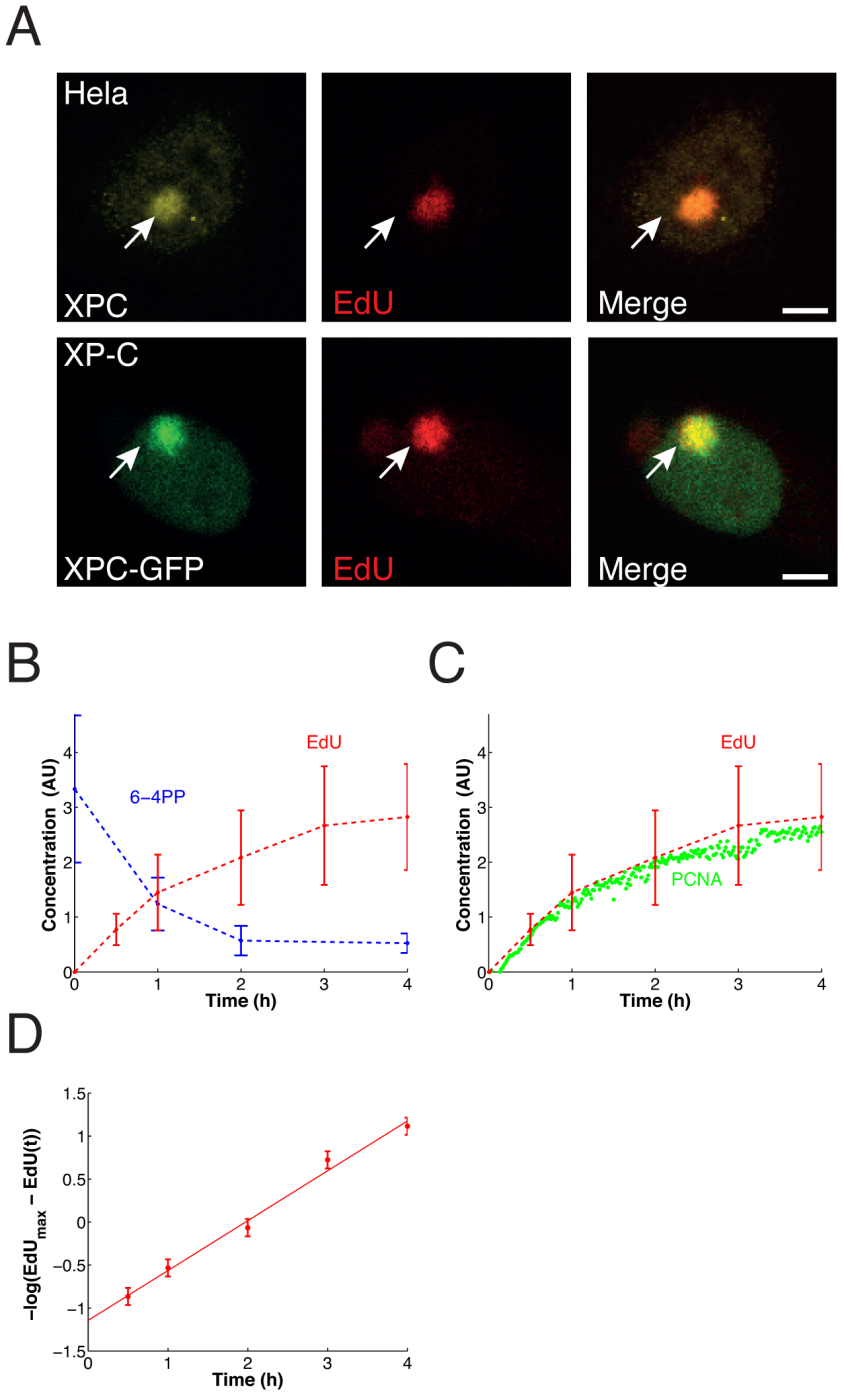
EdU incorporation colocalizes with sites of local damage and reflects repair DNA synthesis quantitatively. (**A**) DNA in HeLa cells (upper panel) or XP–C XPC-eGFP cells (lower panel) was locally damaged by UV irradiation and subsequently incubated for 30 minutes in the presence of 10 µM EdU. Arrows indicate local damage areas as revealed by accumulation of endogenous XPC (upper panel) or stably expressed XPC-eGFP (lower panel); EdU incorporation is a measure for repair DNA synthesis. (**B**) Repair DNA synthesis on sites of local damage as determined by quantitative microscopy coincides with the removal of 6-4PPs measured previously in the same set-up by antibody staining [8]. Graphs display the means ± SD, *n* = 50–70 locally damaged cells per time point for damaged DNA and *n* = 150 locally damaged cells (derived from three independent experiments) per time point for repaired DNA. (**C**) Comparison of increase in EdU incorporation in locally damaged DNA regions with increase in PCNA accumulation as measured previously [8]. (**D**) Plotting the EdU data according to a linearization of Eq. (1) reveals a single-exponential time course.

To quantify the progression of DNA repair and expand our dataset on the dynamics of NER in intact mammalian cells, we determined the kinetics of repair DNA synthesis after locally inflicting UV-damage in cell nuclei [16]. Although DNA lesion removal is often employed as a measure to track repair, it only captures the systems behavior of the pre-incision steps and does not give information about the post-incision processes. To determine the repair kinetics accurately, we induced DNA damage by locally UV-irradiating cells that express an eGFP-tagged version of the lesion-recognition factor XPC in a cell line in which endogenous functional XPC is absent (XP–C [19]) and measured the incorporation of the uridine-analogue, 5-ethynyl-2′-deoxyuridine (EdU), into DNA that was fluorescently labeled after cell fixation [20]. Damaged nuclear areas accumulated XPC-eGFP, and EdU was exclusively present in these areas, consistent with EdU being incorporated through repair DNA synthesis (Fig. 2A). Cells in S-phase, which showed a strong EdU signal throughout the nucleus due to DNA replication, were excluded from our analyses.

To establish the time course of DNA repair synthesis we measured the extent of EdU incorporation at different time points after inflicting UV damage and averaged over multiple cells (Fig. 2B). Of the two main types of UV induced lesions, the 6-4 pyrimidine-pyrimidone photoproducts (6-4 PP) are repaired within a few hours, whereas repair of the cyclobutane pyrimidine dimers (CPD) takes much longer [8], [21], [22]. Indeed, we found that EdU incorporation essentially stops after ∼4 hours, which coincides with the disappearance of the 6-4PPs [8]. Labeling with EdU at different times after UV irradiation indeed showed a continuously declining rate of incorporation and hence of the rate of repair DNA synthesis (Supplementary Fig. S1). This finding is consistent with the progressive disappearance of DNA lesions and demonstrates that EdU availability was not limiting. Moreover, the incorporation of EdU followed the accumulation of PCNA as measured by Luijsterburg et al. [8] in living cells (Fig. 2C). PCNA is required for DNA polymerase function and remains bound to repaired DNA [8], [23], [24]. Taken together, these data establish EdU incorporation as a direct and quantitative measure of DNA repair synthesis in locally damaged cells.

The time course of repair synthesis measured through EdU incorporation is accurately fitted by a single exponential function:
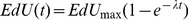
(1)where *EdU*(*t*) and *EdU*_max_ denote the amount of repaired DNA at time *t* and at saturation, respectively (Fig. 2D). The time constant is *λ* = 0.58 (±0.07) h^−1^. This result implies that, despite its molecular complexity, the repair of 6-4PP by NER is effectively a slow first-order reaction with a half-time of 1.2 hours.

### Slow first-order repair kinetics can emerge from interaction of many fast components

**Figure 3:**
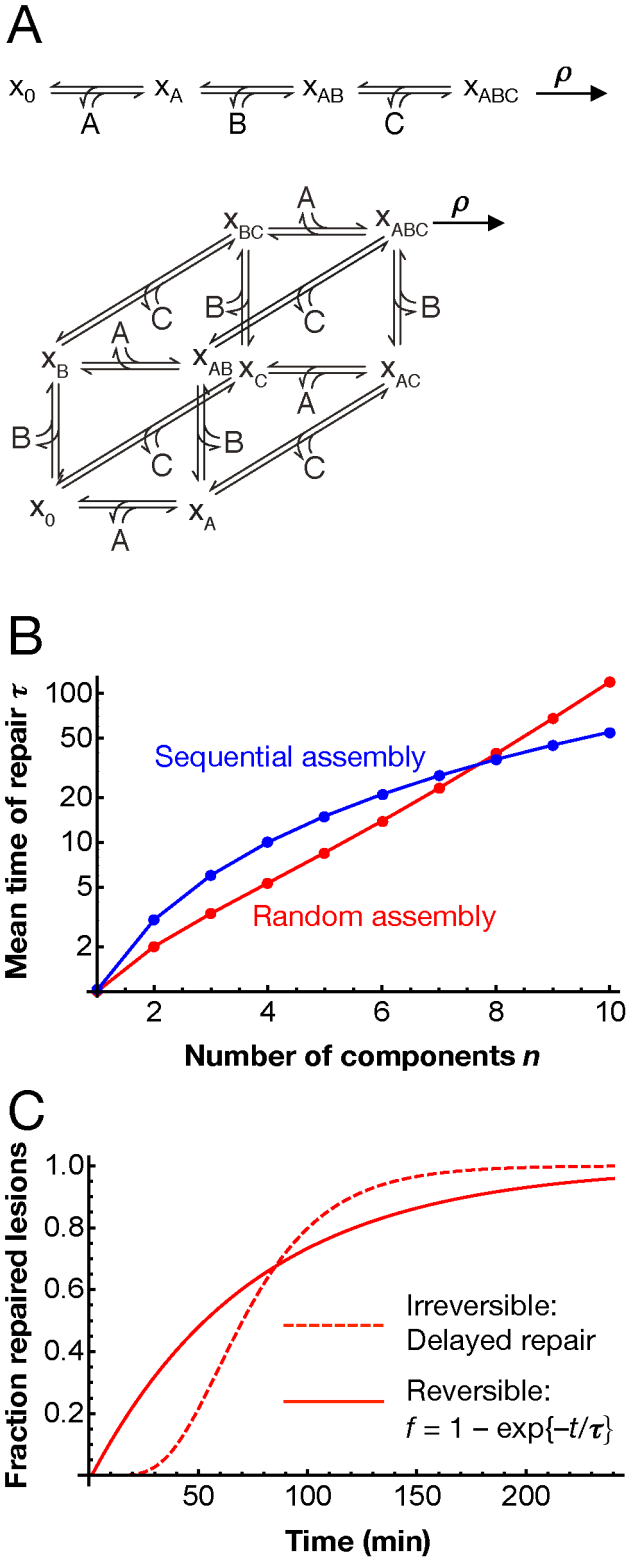
Analytical model of the formation of a catalytic multi-protein complex on DNA. (**A**) Sequential (above) and random (below) assembly schemes for a complex of three components (A, B and C); x_0_ denotes the empty assembly site (e.g., a DNA lesion), x_A_ the assembly site with component A bound etc. The complete complex (x_ABC_) catalyses a reaction (e.g., lesion repair) with rate r. The repair process consists of several such assembly-reaction modules (cf. Fig. 1). (**B**) The mean reaction time *τ* given by Eq. (2) depends on the number of assembling protein components *N* and on the assembly mechanism. Parameters: apparent on-rates *k* equal to off-rates (*k* = *l* = 1 min^−1^), *ρ* = 10 s^−1^ (**C**) In the case of reversible assembly (*k* = *l* = 1 min^−1^, *N* = 9, random mechanism), the formation of the reaction product follows exponential kinetics (solid red line, fitted perfectly by a mono-exponential time course with time constant *τ*). Irreversible assembly (*l* = 0) follows a sigmoidal time course (dashed red line, *N* = 9, random mechanism, on-rate *k* = 0.037 min^−1^ chosen to get the same time constant). The results for a sequential assembly mechanisms are qualitatively similar.

The observed slow first-order kinetics of repair DNA synthesis could be due to a rate-limiting step with a long half-time of the order of an hour. However, there is no likely candidate among the molecular steps of NER for such a slow process [2]. Therefore, we asked whether the first-order characteristics of NER could arise by another mechanism. To gain analytical insight, we first considered a simplified model of repair, with *N* factors assembling into a repair complex and carrying out the repair reaction (Fig. 3A). Under appropriate assumptions, the average time after which a lesion is repaired can be computed as
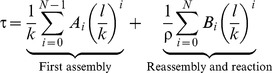
(2)where *k* denotes the pseudo-first order association rate constant of NER factors, *l* the dissociation rate constant of NER factors and ρ the rate of the repair reaction (Supplementary Text S1). The coefficients *A_i_* and *B_i_* depend on whether the repair complex is assembled in a sequential or in a random order. The assumption of common association and dissociation rate constants for all NER factors is motivated by our finding that the core NER factors bind reversibly to a DNA lesion with typical dwell times of the order of ∼1 min [8], taking *k* = *l* = 1 min^−1^ as reference values. Random and sequential assembly of the repair complex give rise to similar average repair times for ten or less assembling components (Fig. 3A); for comparison, global-genome NER in living cells involves 10 core components (cf. Fig. 1B). Remarkably, the repair time is in the experimentally observed hour range for a realistic number of factors, despite the fact that all individual processes (factor binding, dissociation and catalysis of the repair reaction) are much faster. Moreover, with these parameters the model yields a single-exponential time course for the repair of the DNA lesions (Fig. 3B). Further analysis shows that approximately single-exponential kinetics result when the repair factors bind reversibly, whereas high-affinity, near-irreversible binding would lead to a sigmoid time course (Fig. 3C; Supplementary Text S1).

Thus the simplified model of NER indicates that the assembly of a repair complex from rapidly exchanging components naturally generates slow, first-order kinetics of the repair process. This finding implies that the experimentally observed kinetics are an emergent phenomenon of the interplay of many fast components that stochastically distribute the repair of DNA lesions over a broad time period.

### Data-based model of nucleotide-excision repair predicts collective rate control

**Figure 4:**
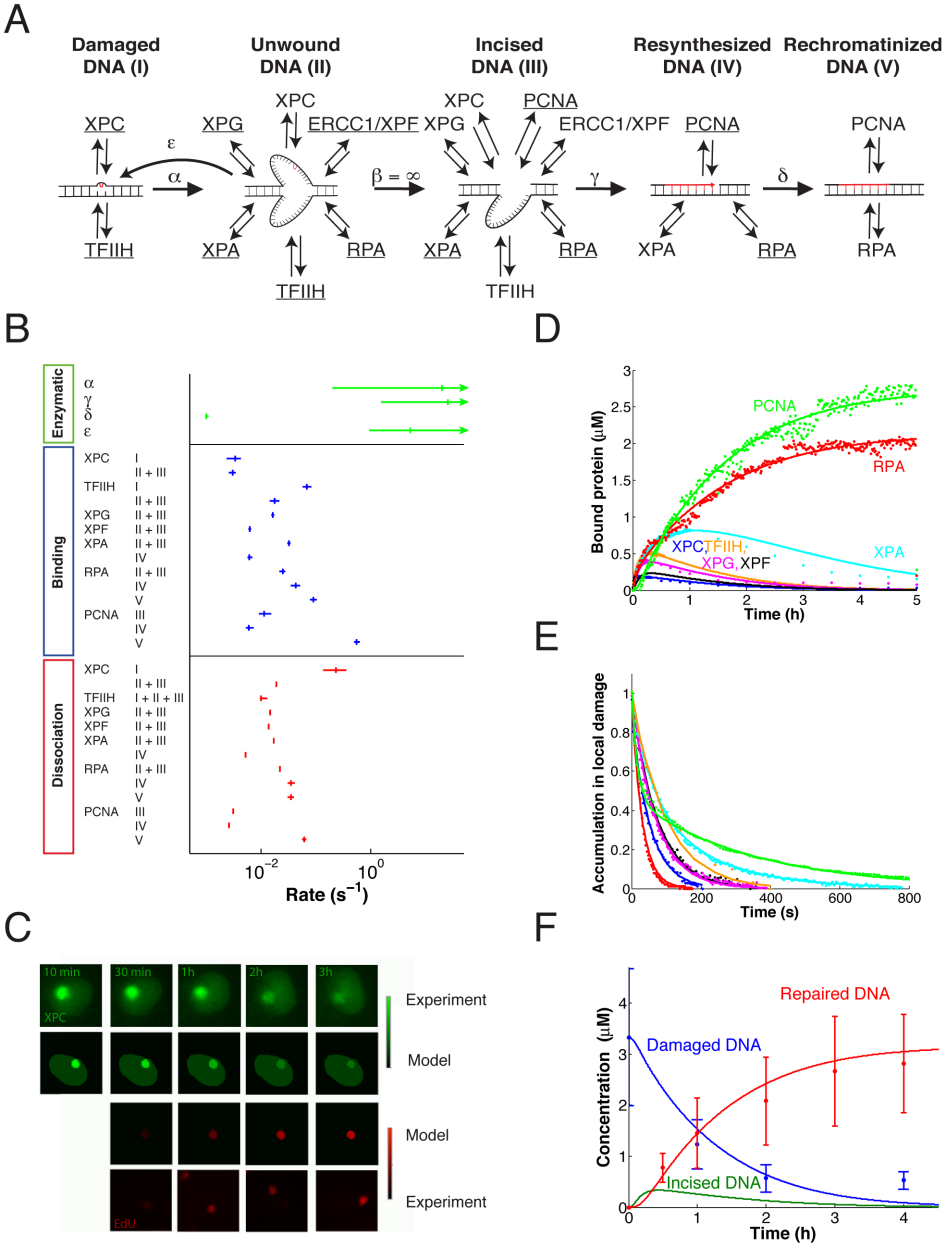
Data-based model of NER reproduces *in vivo* kinetics of the repair proteins and repair reactions. (**A**) The model of NER distinguishes 5 DNA repair intermediates as indicated (I–V) that are interconverted by enzymatic steps: *α*, DNA unwinding; *β*, dual incision; *γ*, re-synthesis; *δ*, re-chromatinization; *ε*, re-annealing of unwound DNA. The repair proteins bind reversibly to the respective repair intermediates as indicated. Underlined proteins together catalyse the transition from one repair intermediate to the next. (**B**) Best-fit values for the model parameters with 95% confidence intervals determined by the profile-likelihood method [25]. The binding rate constants are shown as apparent first-order rate constants obtained by multiplying the fitted *k*_on_ values with the free concentrations of the respective repair factors (taken from [8]). (**C**) Comparison of experimental and simulated time courses for XPC and EdU incorporation. (**D**) Simulated accumulation time courses (lines) of all NER factors in the model compared to experimental data (dots) [8]. (**E**) Simulated FLIP (fluorescence loss in photobleaching) curves (lines) of all NER factors in the model compared to experimental data (dots) [8]. These curves show the short dwell times resulting from individual binding events. (**F**) Computed (lines) and measured (dots with error bars) time courses of the repair intermediates. The simulated time course of the relative amount of the incised DNA intermediate is shown.

To further examine this hypothesis we derived a realistic model of NER based on a large set of quantitative experimental data. The model follows the concept developed by Luijsterburg et al. [8], assuming independent binding of the NER components to the DNA lesion that, transiently forming the appropriate complexes, catalyse the sequence of repair steps. The experimental data consist of the time course of repair DNA synthesis established here and the binding and dissociation rate measurements for all core NER factors (XPC, TFIIH, XPA, XPG, ERCC1-XPF and RPA), as well as PCNA involved in repair synthesis [8] (Fig. 4A). No live-cell imaging data are available for replicative DNA polymerases due to the lack of functional fluorescently tagged constructs; they are assumed to behave in a similar manner as PCNA (which loads the DNA polymerases). Using a standard maximum-likelihood approach for parameter fitting together with the profile-likelihood method to establish parameter bounds [25], we found that these data identify a realistic kinetic model of NER. The present model consists of the equations first derived in [8], with few simplifications to reduce the number of kinetic parameters (in particular, the incision of the DNA lesion has been assumed practically instantaneous once the pre-incision complex has been completely assembled; Supplementary Text S1). The catalytic rates are fast (except for the re-chromatinization rate *δ*). This is seen by the existence of lower bounds on the rate constants of the order of 1 s^−1^ (Fig. 4B). All protein binding and dissociation rate constants are identified within narrow bounds (Fig. 4B) as seen by computing the profile likelihoods (Supplementary Fig. S2). For the numerical values of the parameters see Supplementary Tables S1 and S2.

The model quantitatively simulates the net accumulation of NER factors and the incorporation of EdU (Fig. 4C, D), their behavior in FLIP-based protein dissociation measurements (Fig. 4E) and the time course of repair DNA synthesis (Fig. 4F). The concentrations of NER factors and DNA repair intermediates are given as local concentrations in the damaged area, based on our estimate that the volume associated with the damaged chromatin makes up on average 10% of the total nuclear chromatin per UV-damaged spot (note that in Luijsterburg et al. [8], we used a different normalization by dividing the number of bound proteins with the entire nuclear volume; this resulted in the average nuclear concentrations of bound factors that are tenfold smaller than the –more intuitive – concentrations in local damage given here).

The dissociation constants *K*_d_, which account for the quantitative accumulation of the NER factors at local damage, fall into a rather uniform, biologically realistic range between ∼100 nM (for XPA) and ∼1 µM (for RPA) (Table 1). A notable exception is the initial XPC binding to damaged DNA, which shows a comparatively low affinity (*K*_d_∼9 µM). These quantities are consistent with previous work [8]. However, due to the direct observation of the repair DNA synthesis kinetics, we have now been able to identify practically all model parameters. Importantly, the model implies that the excision of the lesion is followed by repair synthesis without much delay. Therefore, there is only little accumulation of the lesion-excised DNA repair intermediate (Fig. 4F).

**Table 1:** K_D_ values.

Value	XPC	TFIIH	XPG	XPF	XPA	RPA	PCNA
**Damaged DNA**							
K_D_ (µM)	9.35	0.052	NA	NA	NA	NA	NA
	(3.46; 16.09)	(0.044; 0.072)					
**Unwound DNA**							
K_D_ (µM)	0.864	0.204	0.395	2.446	0.147	1.222	NA
	(0.635; 1.006)	(0.163; 0.259)	(0.373; 0.419)	(2.344; 2.596)	(0.138; 0.158)	(1.048; 1.36)	
**Incised DNA**							
K_D_ (µM)	0.864	0.204	0.395	2.446	0.147	1.222	0.388
	(0.635; 1.006)	(0.163; 0.259)	(0.373; 0.419)	(2.344; 2.596)	(0.138; 0.158)	(1.048; 1.36)	(0.319; 0.538)
**Resynthesized DNA**							
K_D_ (µM)	NA	NA	NA	NA	0.236	1.167	0.605
					(0.222; 0.27)	(0.924; 1.521)	(0.531; 0.747)
**Rechromatinized DNA**							
K_D_ (µM)	NA	NA	NA	NA	NA	0.538	0.154
						(0.438; 0.645)	(0.134; 0.182)

NA, not applicable. K_D_ (k_off_/k_on_) values are given for every repair protein and arranged in columns. Reference parameter set and 95% confidence intervals (in parentheses) are shown.

**Figure 5:**
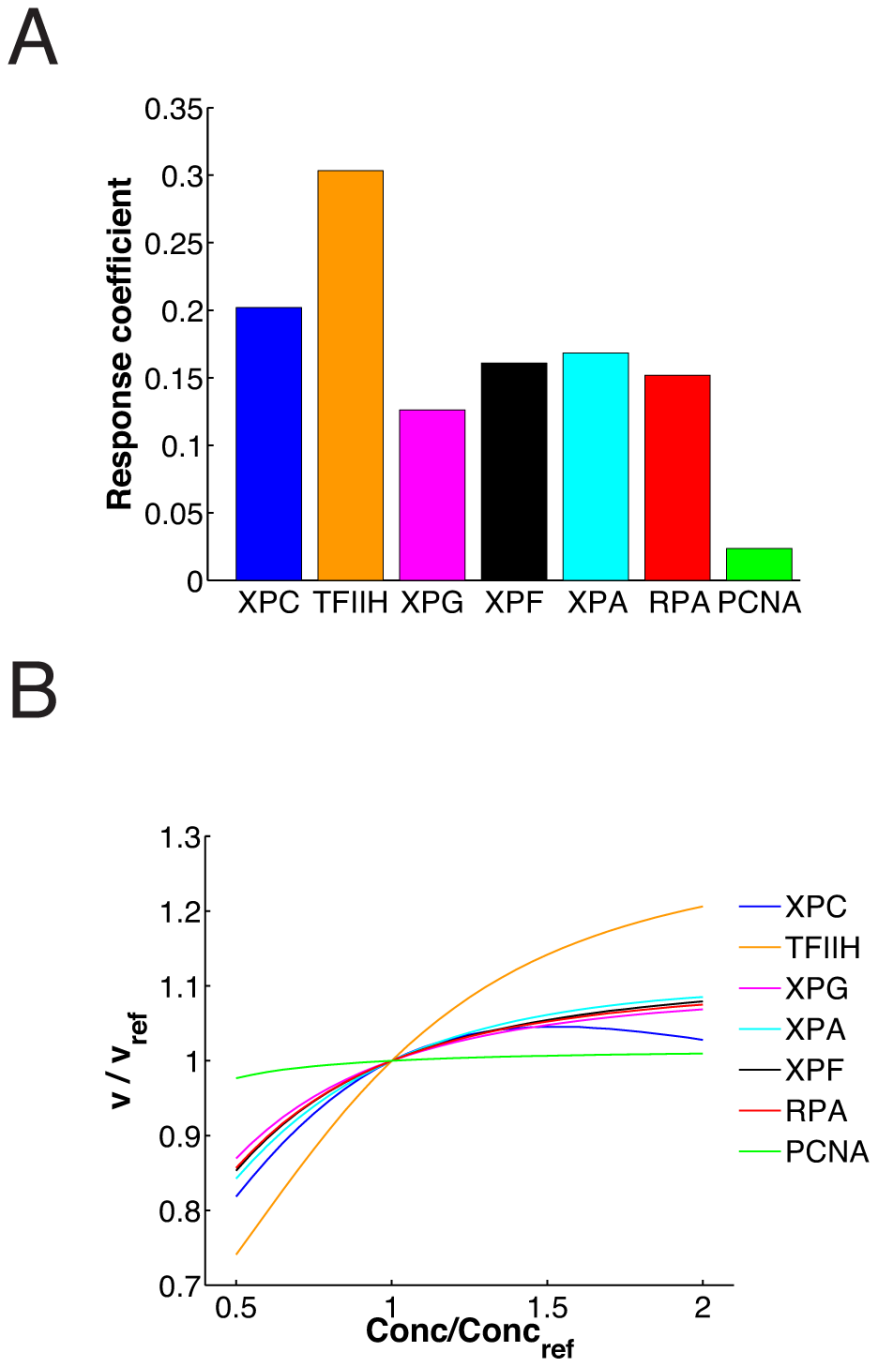
Control over the rate of DNA repair predicted to be small and distributed over all repair proteins. (**A**) Response coefficients for the control of the repair rate by the concentrations of the repair factors. (**B**) Repair rate as a function of concentration changes in individual repair factors.

The narrow confidence bounds on the parameters of the model allow us to make valid computational predictions. We asked whether the kinetics of repair DNA synthesis in this realistic model are indeed controlled by multiple NER factors, as the ‘cartoon’ model (Fig. 3A) suggests. To this end, we evaluated the response coefficients
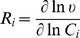
(3)giving the relative change of the repair rate *v* as a function of the relative change in the concentration of the *i*th repair factor *C_i_* (*i* = XPC, TFIIH, …) [26]. All response coefficients have narrow confidence bounds as evaluated by the prediction profile likelihood method [25], implying a well-defined prediction (Supplementary Fig. S3A). The response coefficients are uniformly small (∼0.3 and below, Fig. 5A), and hence there is no singular rate-limiting component distinguished by a high response coefficient. A very similar result is obtained for the control of the dual incision rate (Supplementary Fig. S3B). We also computed the response of the repair rate to large variations in protein concentrations, finding that the response coefficients correctly predict the impact of the individual repair factors (Fig. 5B). The linear approximation (on which the response coefficients are based) yields a reasonable description for about two-fold concentration decreases or increases (corresponding to a knockdown or overexpression experiment), while for very large decreases the repair rate drops eventually to zero (corresponding essentially to a gene knockout).

In summary, these findings imply that the individual repair factors have similar, small control on the repair rate. Thus the model predicts that the repair rate is collectively controlled by all NER components and, therefore, robust against variations in the concentration of any individual factor.

### Exploiting natural variability in protein expression to quantify rate control

**Figure 6:**
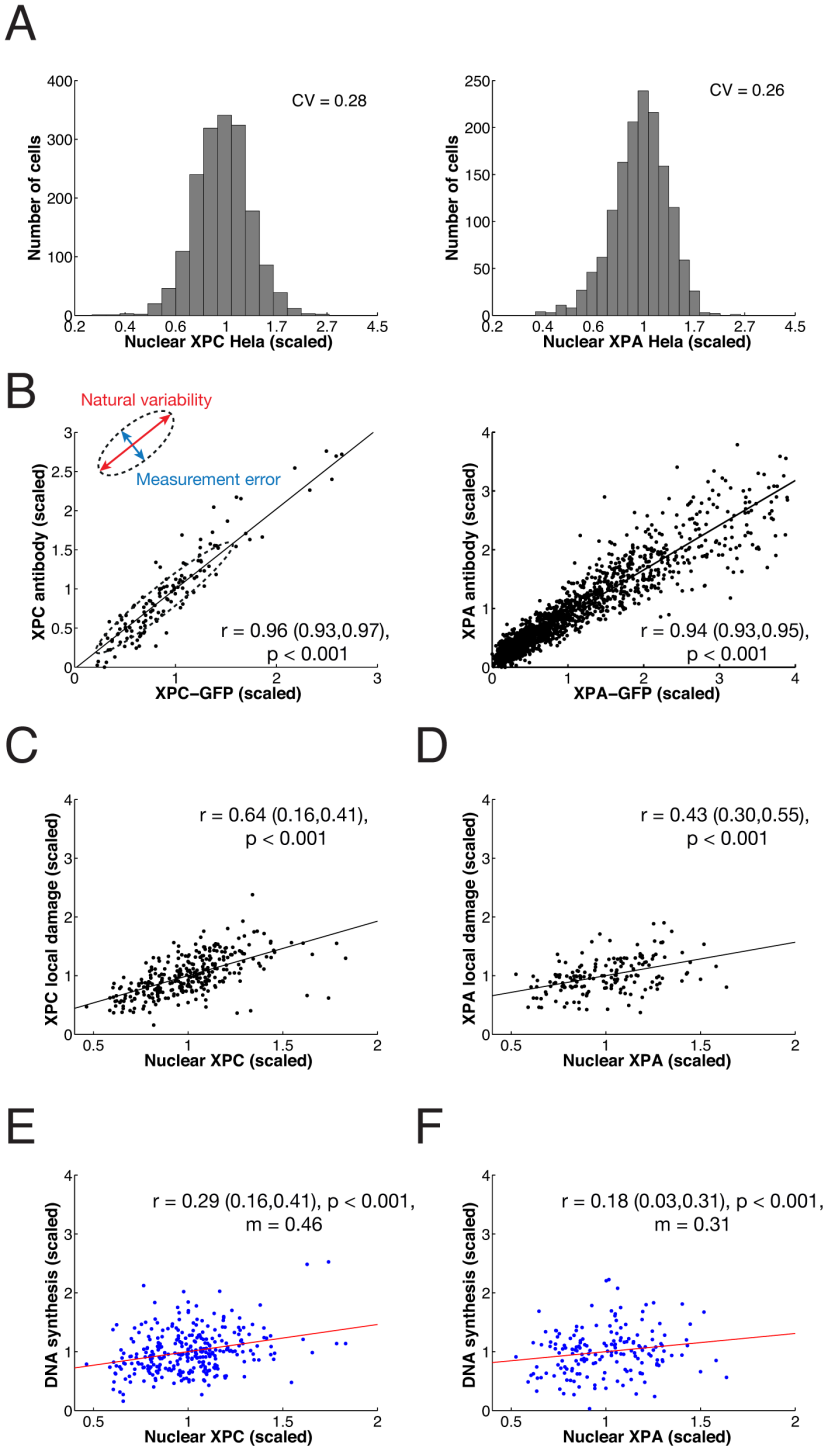
Natural expression variability of the repair factors XPC and XPA has small effect on DNA repair synthesis. (**A**) Frequency histograms of endogenous XPC (*n* = 1727) and XPA (n = 1462 cells) concentration in HeLa cells. (**B**) Scatter plot of XPC-eGFP fluorescence in XP–C XPC-eGFP cells against an antibody recognizing XPC (left, *n* = 332 cells) and eGFP-XPA fluorescence in XP–A eGFP-XPA cell line against an antibody recognizing XPA (right, n = 2142 cells) as determined by quantitative (immuno) fluorescence microscopy. (**C**) Scatter plot of endogenous XPC nuclear concentration versus endogenous XPC concentration in DNA damaged areas 30 minutes post-irradiation. (**D**) Endogenous XPA nuclear concentration versus endogenous XPA concentration in damaged areas 60 minutes post irradiation. (**E**) Scatter plot of endogenous XPC nuclear concentration versus repair DNA synthesis as measured by EdU incorporation on local damage 30 minutes post-irradiation (*n* = 303 in three independent experiments). (**F**) Scatter plot as in (**E**), but for XPA (n = 170 cells). Cells were analysed 60 minutes after inflicting UV damage, i.e. at maximum XPA accumulation. 95% confidence bounds of all correlation coeffiecients *r* were estimated by non-parametric bootstrap and are given in brackets.

To determine the effect of NER factors on repair synthesis, we focused on the lesion recognition factor XPC and the component of the pre-incision complex XPA. We observed considerable variability in the endogenous expression levels of both proteins in individual cells (Fig. 6A), with coefficients of variations (*CV*) of ∼0.3. However, the theoretical analysis of rate control predicted that across this range of natural variability (roughly from about one half of the average expression level to twice the average; cf. Fig. 5B), the repair rate of 6-4PP should be affected only moderately. We asked whether rate control could be quantified experimentally by exploiting the natural expression variability of the NER factors.

To this end, we first examined the accuracy of XPA and XPC antibody staining. In human XP–C and XP–A fibroblasts stably expressing XPC-eGFP and eGFP-XPA, respectively [19],[27], we observed a high correlation between the eGFP signal and immunofluorescent labeling (Fig. 6B). Fitting an error ellipse to these data (Supplementary Text S1), we estimated a relative measurement error of antibody labeling of 11%, showing that the technique is suitable for quantitation of nuclear XPC and XPA concentrations. We then examined whether the nuclear concentration of a repair factor is correlated with its accumulation at UV-induced DNA lesions and with the rate of repair DNA synthesis. Thirty minutes after local UV irradiation we observed a linear relationship between the nuclear concentration of XPC and the concentration of accumulated XPC in the damaged spot (Fig. 6C). This result demonstrates that the DNA lesions are not saturated with XPC, so that a higher free XPC concentration in the nucleus could accelerate the repair rate. The same holds true for XPA (Fig. 6D) and has previously been shown for the endonuclease XPG [8]. Therefore, we asked next whether increased accumulation of the repair factors accelerated repair. The measurement of the concentration of XPC or XPA in the nucleus simultaneously with EdU incorporation showed a significant positive correlation (Fig. 6E and F, respectively). However, the dependence of EdU incorporation on the concentration of either factor was weak, as seen by the small slopes of the regression lines. Remarkably, the slopes were in the range predicted by the response coefficients of XPC and XPA for the repair rate. These data suggest that the repair rate is largely robust against natural variability of XPC and XPA concentration, supporting the prediction of the sensitivity analysis of the model (cf. Fig. 5).

**Figure 7:**
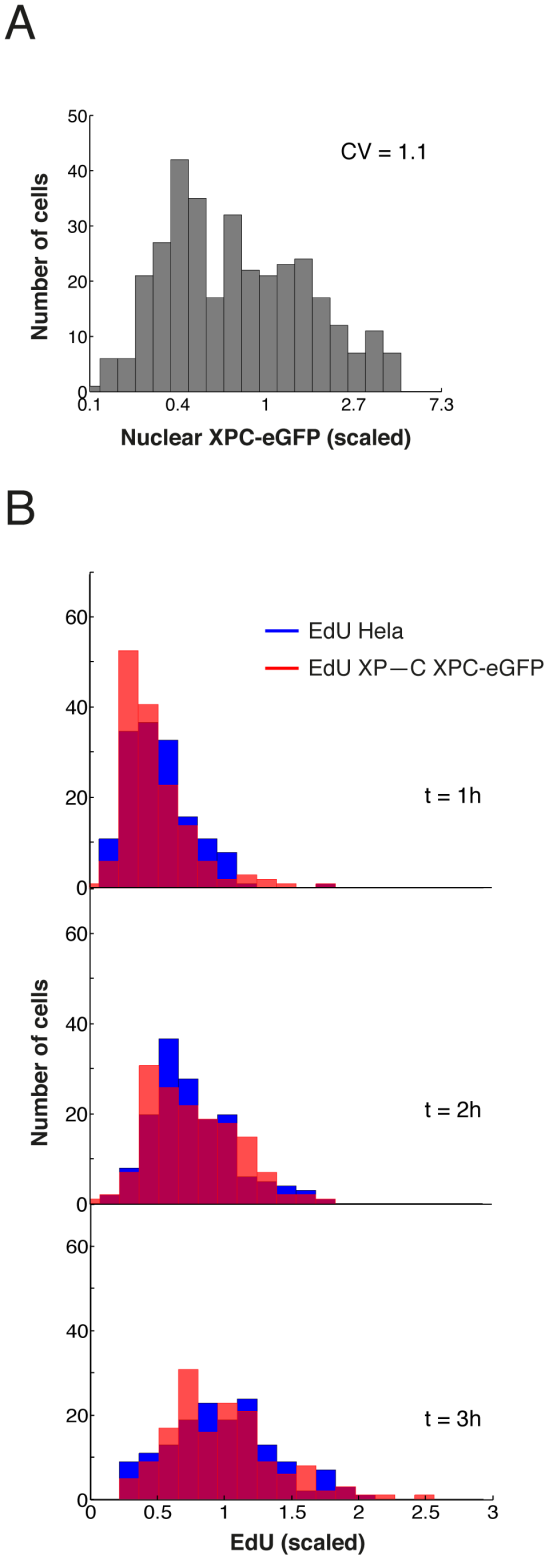
Variability of NER in XPC-eGFP complemented XP–C patient cell line is comparable to native NER. (A) Variability of XPC-eGFP (left, n = 332) concentration in stably transfected XP–C. (B) Distribution of incorporated EdU in local damages measured in XP–C XPC-eGFP (red) or HeLa (blue). n = 154 cells per time point per cell line.

The finding of robustness of repair rate is further strengthened by the observation that in XP–C cells complemented with XPC-eGFP, the expression levels of the fluorescent protein expressed from the stably integrated transgene varied more strongly than the endogenous XPC protein levels in HeLa cells (CV∼1, with peak levels in a sizeable number of cells reaching 3–4 times the average), yet EdU incorporation after UV damage followed very similar dynamics in both cell types (Fig. 7).

**Figure 8:**
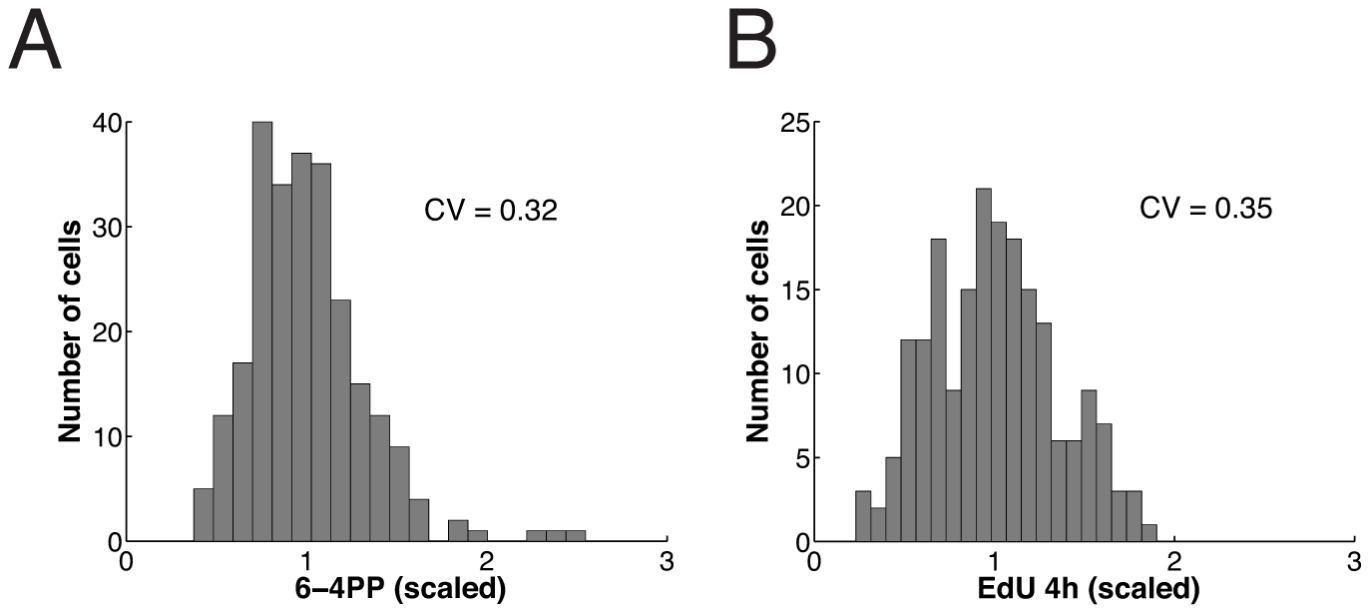
Initial distribution of inflicted lesions (6-4 photoproducts) and final distribution of EdU incorporation match. (**A**) XP–C XPC-eGFP cells were locally irradiated and fixed immediately. The distribution of inflicted 6-4PP lesions was determined by immunostaining followed by scoring of the immunofluorescence signal in *n* = 250 cells from five experiments (**B**) XP–C XPC-eGFP cells were locally irradiated and cultivated for 4 hours in the presence of EdU before fixation. Distribution of EdU incorporation was plotted based on measurements derived from *n* = 198 scored local damages derived from three independent experiments.

The considerable scatter in Figures 6E and F indicates that there are further sources of cell-to-cell heterogeneity. We quantified the amount of inflicted DNA lesions (6-4PP) after UV damage and incorporated EdU after repair of 6-4PPs is completed. Both quantities show variability between individual cells (Fig. 8A,B). That the initial distribution of 6-4PP and the final distribution of EdU incorporation are of very similar width shows the consistency of the two measurements. These data indicate that the amount of inflicted DNA lesions contributes to the observed cell-to-cell variability in repair rate.

### Variable expression levels of NER factors and inflicted DNA lesions quantitatively account for the distribution of repair rates

**Figure 9:**
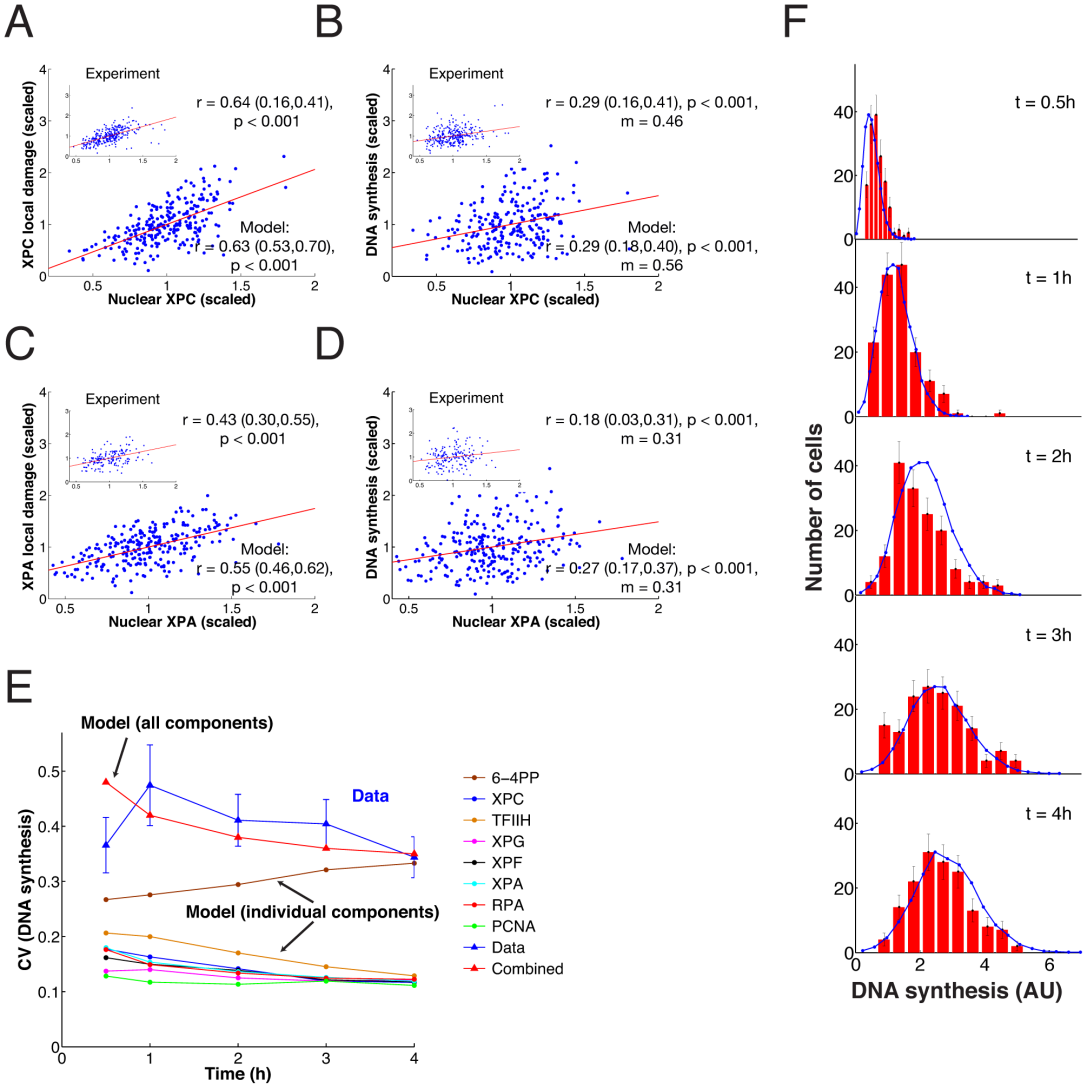
Variable expression levels of NER factors and inflicted DNA lesions quantitatively account for the distribution of repair rates. Comparison of modeled and measured correlation of nuclear XPC concentration and XPC accumulation in the locally damaged region (**A**) and between nuclear XPC concentration and DNA synthesis (**B**). Red lines represent linear regression with correlation coefficient *r* and *p*-value. (**C**) and (**D**) as in (A) and (B) but for XPA. (**E**) Simulated variability of the DNA repair kinetic under the influence of a single variable protein (dots) and of all variable components (triangles). For comparison the experimental *CV* is shown at five time points and error bars obtained with nonparametric bootstrap. (**F**) Time evolution of distribution of EdU incorporation: measured (red bars) versus simulated (blue lines). 95% confidence bounds of all correlation coeffiecients *r* were estimated by non-parametric bootstrap and are given in brackets.

How do these two sources of cell-to-cell variability that we identified experimentally – concentration differences in NER factors and number of inflicted DNA lesions – control the rate of repair? To analyse this quantitatively we first simulated the simultaneous effect of both sources with the model. To simulate an individual cell we drew randomly an amount of 6-4PP from the measured distribution (cf. Fig. 8A) and equipped the cells with random amounts of all NER factors according to lognormal distributions with typical width (*CV_i_* = 0.25; cf. Fig. 6B) and the experimentally determined mean values [8]. Repeating this for several hundred cells (as in the experiments) we found that the simulated relation between nuclear XPC concentration and XPC accumulated in local damage agreed remarkably well with the measured data (Fig. 9A); the same held true for the relation between nuclear XPC and extent of DNA repair synthesis at 30 min after irradiation (Fig. 9B). Quantitative agreement was also found for the experimental data and the simulated relations between nuclear XPA concentration and XPA accumulated in local damage (Fig. 9C), as well as extent of DNA repair synthesis at one hour after irradiation (Fig. 9D).

These findings indicate that the variable concentrations of NER factors and inflicted DNA lesions explain the observed cell-to-cell variability in repair DNA synthesis. Therefore, we asked whether the individual contributions of the concentration distributions of the repair factors to the distribution of the repair rate ‘sum up’ according to the response coefficients predicted by the model. According to the law of propagation of uncertainty we have approximately
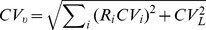
(4)where *CV_v_*, *CV_i_* and *CV*_L_ denote the coefficients of variation of the distributions of repair rate (*v*), repair factors (*i* = 1,2,…) and initial amount of lesion (index L) (Supplementary Text S1). Assuming that all *CV_i_* = 0.25, as measured for XPC and XPA, and *CV*_L_ = 0.32, we find with the response coefficients of the model *CV_v_* = 0.34. This value compares well with the experimentally measured *CV* of the distribution of EdU incorporation at 30 min, 0.37, where the difference might be due to the fact that a few further factors (DNA polymerases and ligases) are involved in repair but have not been accounted for explicitly in the model.

To dissect the contribution of the individual factors to the overall variability, we computed the effect of heterogeneity in only one factor on repair synthesis. The initial distribution of 6-4PPs has the strongest impact but, clearly, does not account for the entire observed variability, particularly at earlier time points (Fig. 9E, observed: blue triangles). As expected from the size of the response coefficients, the cell-to-cell variability in the expression levels of the individual NER factors have all comparable and rather small effect (Fig. 9E, model individual factors: dots). Adding up the contributions from all factors closes the ‘variability gap’ left between the distribution of 6-4PP and repair synthesis (Fig. 9E, model all factors combined: red triangles). Indeed, the computed time evolution of the distribution of EdU incorporation largely matches the measured one (Fig. 9F).

To summarize, we uncovered two sources of cell-to-cell variability in the rate of NER: (i) the amount of inflicted DNA lesions and (ii) the expression of NER factors. The quantitative impact of these two parameters on the cell-to-cell variability of the repair rate is consistent with the robustness of the repair rate against concentration fluctuations in individual repair factors.

## Discussion

In this paper we have quantified the rate of DNA repair by the nucleotide-excision repair pathway in relation to the behavior of the individual NER components. The experimentally observed slow, apparent first-order kinetics of repair agree with the prediction from mathematical modeling of NER. Importantly, the model indicates that these kinetics are not due to a rate-limiting step in the pathway but, rather, are an emergent phenomenon of the rapid interplay of many transiently interacting components. An important functional consequence of this kinetic design is that the control of the repair rate is shared by all repair factors. This collective rate control implies that the rate is robust against concentration variations in individual repair proteins. Exploiting the natural variability of protein expression in mammalian cells, we have provided experimental support for this model.

Early measurements of repair rate by radionucleotide incorporation displayed two components of NER, one being completed in several hours and the other lasting much longer [28]. As indicated by the removal kinetics of the two main types of DNA lesions, 6-4PP and CPD, the first component represents the repair of 6-4PPs, which is practically complete within four hours, and the second component is CPD repair, which is still negligible after eight hours [8]. Here we monitored the first component, 6-4PP repair, via EdU incorporation and showed that it follows first order-kinetics with half-time of 1.2 hours. This long time scale (hours) is in sharp contrast with the results of imaging the dynamics of NER proteins in living cells. These latter studies showed that all protein-substrate interactions are transient and occur on a time scale of seconds to a few minutes [8], [12]–[15], [19], [27], [29]. The individual catalytic steps (DNA unwinding, lesion excision and repair synthesis) are also thought to be fast. Indeed, a recent direct measurement has shown that DNA repair synthesis and ligation in bacteria take seconds [30]. The present work proposes a resolution to the conundrum that the overall rate of lesion repair is nevertheless slow by showing how the repair kinetics emerge as a systems property from the interplay of the underlying molecular processes.

Our mathematical analysis shows that the reversibility in the binding of multiple proteins to the DNA lesion, and subsequent repair intermediates, is the key property that makes DNA repair an apparent first-order process. Intuitively, the broad distribution of repair times results from the fact that protein binding and dissociation events will be iterated a variable number of times before an active repair complex is formed. This is in contrast to a sequence of irreversible binding steps, which will create sigmoidal kinetics with an even sharper delay with increasing numbers of assembling components. When comparing with the degree of reversibility, the order of component assembly (random, sequential or combination of the two extremes) is less relevant for whether the repair kinetics will be first-order (exponential) or sigmoidal. Fast exchange of individual factors is a ubiquitous property of chromatin-interacting proteins in transcription, chromatin remodeling and repair [1], [31]. It is therefore tempting to speculate that the slow time scales of transcriptional bursting in mammalian cells (tens of minutes to hours; [32], [33]) also arise through the reversible assembly of large macromolecular complexes.

High molecular off-rates have been suggested to be a general feature of self-organizing systems in the cell as they allow for efficient exploration of an assembly landscape and selection of a functional steady state [34]. In the case of chromatin-associated machineries; specificity and regulatability will both benefit from reversible binding of repair proteins or transcription factors [8]–[10], [35].

Based on comprehensive experimental data, we have here developed a predictive mechanistic model of NER. Progressing beyond previous models [8], [36], [37], this appears to be the first model of a DNA-associated process for which the model parameters could be identified (i.e., parameter values uniquely assigned with narrow confidence bounds) from the experimental data. Achieving this required a number of simplifications, including the neglect of protein-protein interactions and the explicit consideration of most but not all core NER factors, selecting those for which live-cell imaging is currently possible. Judged by the rigorous statistical criterion of identifiability, the current model provides a concrete picture of what can be said *quantitatively* about the regulation of NER. Adding further mechanistic detail will require appropriate quantitative measurements to evaluate their impact on systems properties such as repair rate, robustness against protein-expression noise and fidelity of lesion recognition. We expect that the general dynamic behavior of the model will prove robust with respect to the addition of further molecular detail and components because the dynamics are already produced in a qualitative manner by a simplified ‘cartoon’ model of repair (see Fig. 3). Our work might also provide a useful reference for mathematical models of transcription, which so far have been parameterized on the basis of FRAP data without studying parameter identifiability [9], [17], [18].

The NER model makes two interesting predictions on the function of the repair pathway in living cells. First, lesion excision and repair synthesis are tightly coupled, preventing undue accumulation of single-stranded DNA after excision of the lesions. In this kinetic aspect, *in vivo* NER appears to differ from *in vitro* studies that have found a delay before repair synthesis [38], [39]. Preventing the accumulation of single-stranded repair intermediates in the cells that are vulnerable to being cleaved is a plausible functional objective and, as shown here, is consistent with data obtained on intact cells. Second, there is no singular rate-limiting step in the repair pathway. Instead, all repair factors control the repair rate collectively, with the contribution of each individual factor being rather small. Thus the control of the DNA repair rate has similarity to the control of flux in metabolic pathways which has also frequently found to be shared by several enzymes [26]. It is important to note that rate control is a kinetic property that is not linked to the order at which the factors bind. Thus the recognition factor XPC that binds first to a DNA lesion and XPA, which binds much later, have very similar rate control. In particular, our results show that the functioning of the NER pathway is robust against the natural fluctuations of protein concentrations in the cell.

The model can also be used to rationalize the effect of larger perturbations in the concentrations of NER factors. In particular, strong reduction of any factor participating in damage recognition and excision should compromise function (e.g., repair rate) in a similar manner. Indeed, reduction in XPA or XPC concentration leads to decreased cell survival and/or decreased lesion removal [40]–[42]. Increased expression levels of ERCC1 and XPA have been correlated with increased resistance of certain tumors to cisplatin, which induces DNA interstrand crosslinks specifically removed by NER [40]–[44]. It would be interesting to investigate whether other NER factors are also elevated under these conditions, as a collective rate control mechanism would suggest, or whether XPA or ERCC1 have particularly high control (e.g., through low endogenous expression level) in the cell types and conditions studied.

The mechanism that produces collective rate control in the NER model is not specific to this repair pathway, but should equally apply to the assembly of other chromatin-associated molecular machineries. Indeed, a quantitative study of the interferon-**β** gene transcription has shown that the overexpression of five out of six transcription factors needed to activate transcription had a significant effect on the probability of transcription [45]. Very similar results have recently been obtained for the regulation of expression of the cytokine IL-2 [46]. These experimental findings support a collective process of rate control also for transcription.

## Materials and Methods

### Cell culture

XPC-deficient XP4PASv cells (XP–C cells) stably expressing XPC-eGFP [19] and XPA-deficient XP20SSv cells (XP–A cells) stably expressing eGFP-XPA [27] were maintained in a 1∶1 ratio of F10∶DMEM supplemented with 5% FBS (Gibco) medium, glutamine and penicillin/streptomycin. HeLa cells were grown in DMEM medium supplemented with 5% FBS, glutamine and penicillin/streptomycin. All cells were kept in an incubator at 37°C incubator containing 5% CO_2_ for cultivation and in an incubator without CO_2_ after UV-irradiation.

### UV irradiation to inflict local damage

Local irradiation was performed as described by Moné *et al.* [16]. Briefly, cells were grown to confluence on uncoated 24 mm glass coverslips. Before UV-irradiation cells on coverslips were briefly incubated with pre-warmed microscopy medium (137 mM NaCl, 5.4 mM KCl, 1.8 mM CaCl_2_, 0.8 mM MgSO_4_, 20 mM D-glucose and 20 mM HEPES, pH 7.0) at 37°C. Immediately upon removal from the incubator cells were overlaid with a mask, containing pores with 5 µm diameter, minimizing the distance between mask and cells. Local irradiation was performed with a dose of 100 J/m^2^ of UV-C with a fluency of 3.85 W/m^2^, as measured at 254 nm with an SHD 240/W detector connected to an IL 1700 radiometer (International Light), unless stated differently. Cells were incubated in microscopy medium in an incubator without CO_2_ at 37°C as indicated in the text.

### EdU incorporation analysis

Prior to UV-irradiation cells on coverslips were briefly incubated in microscopy medium supplemented with 10 µM EdU (5-ethynyl-2′-deoxyuridine, Life Technologies). After irradiation, cells were incubated in medium in the continuous presence of 10 µM EdU. After incubation for the desired time cells were fixed in PBS containing 4% PFA (pH 7.0), permeabilized by incubation with PBS/0.5% Triton X-100 and processed and fluorescently labeled following the manufacturer's instructions (Invitrogen). Briefly, coverslips were incubated with a proprietary Life Technologies reaction mix leading to copper-catalyzed covalent addition of an AlexaFluor-555 fluorophore to incorporated EdU. Subsequently, coverslips were washed twice with PBS and, if necessary, processed for immunolabeling as described below. After processing coverslips were rinsed with sterile ultrapure water, and mounted with Vectashield (Vectorlabs) mounting reagent for microscopy.

### Immunolabelling

Locally irradiated cells were fixed in PBS/4% PFA for 10 minutes, washed 3× with PBS and permeabilized with PBS/0.5% Triton X-100 for 10 minutes. Immediately before 6-4PP immunolabelling cells were incubated with 0.1 M HCl for 10 minutes. Cells were washed 3 times with PBS/3% BSA and incubated with Rabbit anti-XPC (X1129, Sigma-Aldrich) and/or Mouse anti-64PP (64M-2, Cosmo Bio Co, Tokyo, Japan) dissolved in PBS/3% BSA for one hour at room temperature in the dark. Antibody was removed by washing 3 times with PBS/3%BSA and coverslips were incubated with donkey anti-rabbit Cy5 or Cy3 (711-175-152/711-165-152, Jackson Immunoresearch), and/or donkey anti-mouse Alexa488 (715-545-150, Jackson Immunoresearch) and DAPI for one hour at room temperature in the dark. Coverslips were washed 3 times with PBS/3% BSA, rinsed with water and mounted on slides with Vectashield (Vectorlabs) mounting medium before observation. For XPA detection a combination of rabbit anti-XPA (X1254, Sigma-Aldrich) and donkey anti-rabbit Alexa647 (711-605-152, Jackson Immunoresearch) was used, and nuclei were counterstained with Draq5 (Biostatus LTD).

### Microscopy

All microscopic analyses were performed on a LSM510 (Zeiss Inc.) microscope with a fully open pinhole, equipped with the following lasers with the indicated wavelengths; Argon ion1 (364 nm), Argon ion2 (488 nm), Helium-Neon1 (543 nm) and Helium-Neon2 (633 nm). Slides were observed with either a 63× Plan-Apochromat NA 1.40 NA or a 63× Plan-NeoFluar NA 1.25 lens. Images were recorded by Zeiss software in multi-track mode. All images were recorded with 12-bit dynamic range (bits per pixel). The photomultiplier settings were corrected for each channel to ensure image detection within linear detection range.

### Image analysis

All image analyses was done by using ImageJ software (NIH, Bethesda, MD). For indirect immunofluorescence experiments, values were normalized per experiment before comparison and analyses. Nuclear concentrations were measured by thresholding nuclei manually or, when possible, automatically. The mean background outside the nuclei was subtracted to correct for background. XPC and XPA intensities on local damage were determined by manually thresholding local damages; these intensities were background-corrected by subtracting the intensity in a similarly-sized undamaged portion of the nucleus. EdU incorporation was determined by manual thresholding of local damages and intensity measurements. Background correction was performed by subtracting the intensity of a similarly-sized area in the same nucleus. Raw values from different experiments were pooled and plotted directly (distributions) or scaled and plotted. The amount of inflicted DNA lesions was determined by measuring total intensity on local damage after antibody staining. Values were background-corrected by subtracting the mean background intensity outside local damage. Data was scaled per experiment (n = 5), pooled, and plotted. Different antibody concentrations were used to determine antibody linearity; similar CV values were obtained with all concentrations used. Figures and statistical analysis were done in MatLab (Mathworks, August 2010).

### Mathematical modeling

The mathematical models (analytical model and detailed, data-driven model) are based on balance equations for the abundance of protein complexes assembling on the different states of the DNA template (ordinary differential equations), as specified in the Supplementary Text S1. Parameter fitting and simulations were done with Matlab code supplied with this paper. Details on error estimation in Fig. 6B can be found in the Supplementary Text S1.

## Supporting Information

Figure S1 **The rate of EdU incorporation in local damage declines over time.** Irradiated XP–C XPC-eGFP cells were treated with EdU for different time intervals post-irradiation. EdU intensities on local damage were determined and plotted as distributions. Intervals are shown as follows; (A) 15 minutes pre-irradiation to 60 minutes post-irradiation, (B) 45–120 minutes, (C) 105–180 minutes, (D) 165–240 minutes and (E) 225–300 minutes. (F) Line plot of experimental means derived from the distributions shown in (A–E). Error bars denote. SD; n = 50 cells per experiment.
(TIF)

Figure S2 **Profile likelihood estimates yield confidence bounds for the model parameters.** PLE and 95% confidence interval (horizontal red line) of binding and dissociation parameters for damaged DNA (A), unwound and incised DNA (B), resynthesized DNA (C), rechromatinized DNA (D) and all catalytic constants (E).
(TIF)

Figure S3 **Repair-rate response coefficients are identifiable and show a similar distribution as incision-rate responds coefficients.** (A) Prediction profile likelihoods and 95% confidence bounds for all 7 repair factors. (B) Response coefficients for the control of the incision rate by the concentrations of the repair factors.
(TIF)

Table S1 **Values of binding and dissociation rate constants.** NA, not applicable. k_on_, k_off_ and K_D_ (k_off_/k_on_) values are given for every repair protein and arranged in columns. Reference parameter set and 95% confidence intervals (in parentheses) are shown. Nuclear concentration (in micromolars) of NER factors are based on previously described data [8].
(PDF)

Table S2 **Values of the enzymatic rate constants.** Reference parameter set and 95% confidence intervals (in parentheses) are shown. In case of practical non-identifiability only the lower confidence bound is given.
(PDF)

Text S1 Mathematical modeling and analysis.
(PDF)
