# Supplementary figures and images for: Robustness of DNA Repair through Collective Rate Control
Source: PLoS Comput Biol. 2014 Jan 30;10(1):e1003438. doi: 10.1371/journal.pcbi.1003438 (PMC3907289; doi:10.1371/journal.pcbi.1003438)

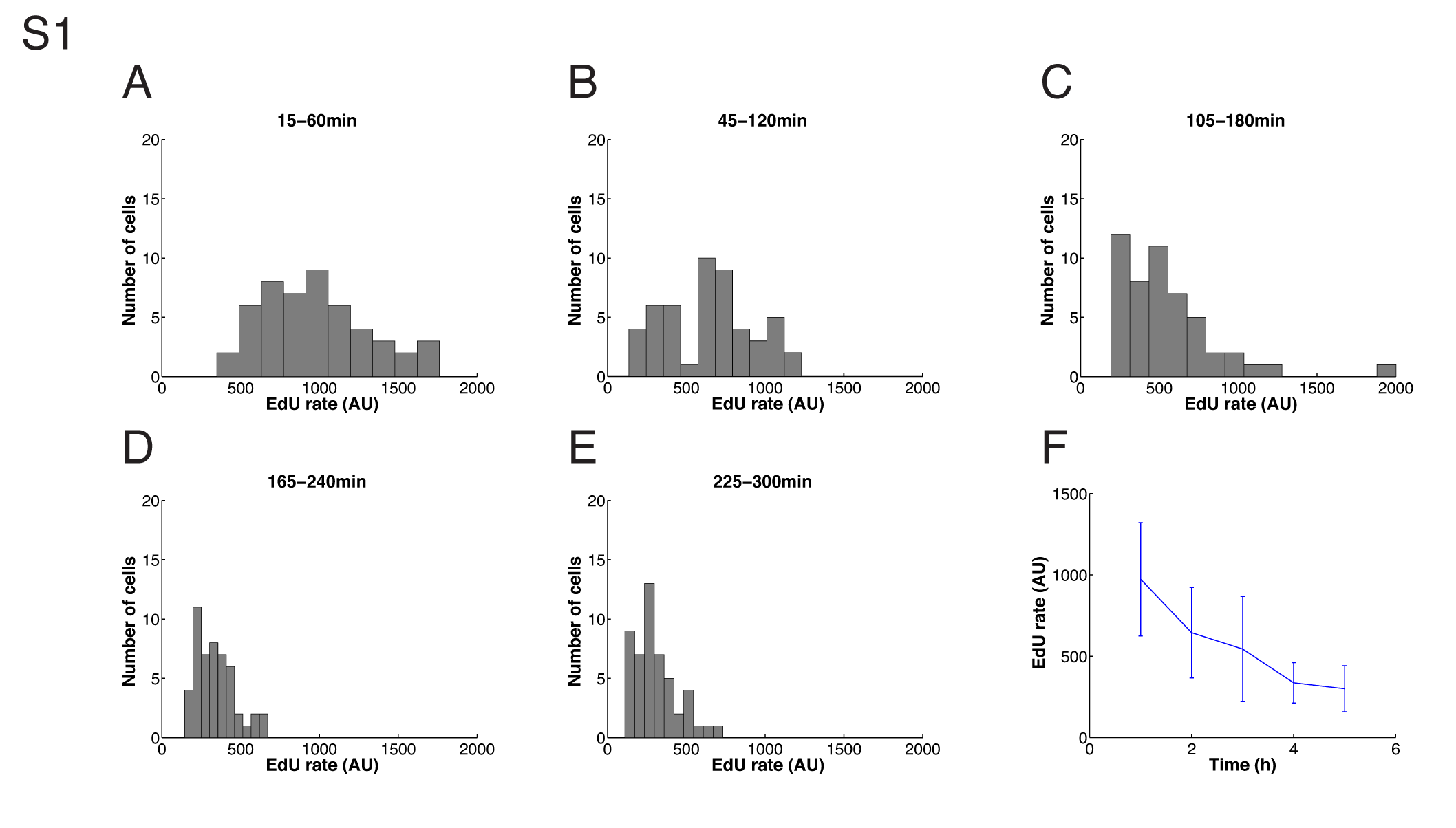

Supplement: Figure S1 — The rate of EdU incorporation in local damage declines over time. Irradiated XP–C XPC-eGFP cells were treated with EdU for different time intervals post-irradiation. EdU intensities on local damage were determined and plotted as distributions. Intervals are shown as follows; (A) 15 minutes pre-irradiation to 60 minutes post-irradiation, (B) 45–120 minutes, (C) 105–180 minutes, (D) 165–240 minutes and (E) 225–300 minutes. (F) Line plot of experimental means derived from the distributions shown in (A–E). Error bars denote. SD; n = 50 cells per experiment. (TIF) [file pcbi.1003438.s001.tif]

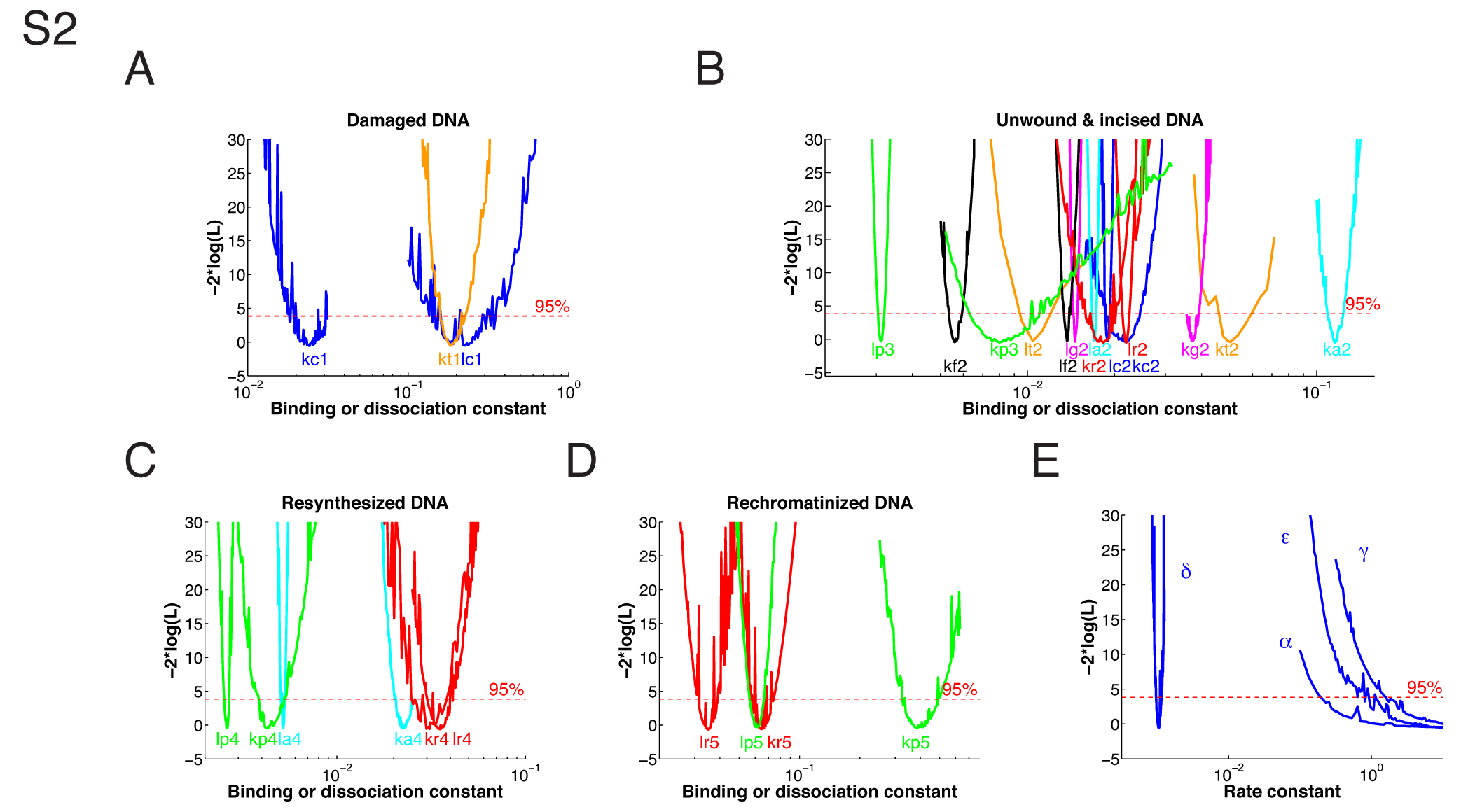

Supplement: Figure S2 — Profile likelihood estimates yield confidence bounds for the model parameters. PLE and 95% confidence interval (horizontal red line) of binding and dissociation parameters for damaged DNA (A), unwound and incised DNA (B), resynthesized DNA (C), rechromatinized DNA (D) and all catalytic constants (E). (TIF) [file pcbi.1003438.s002.tif]

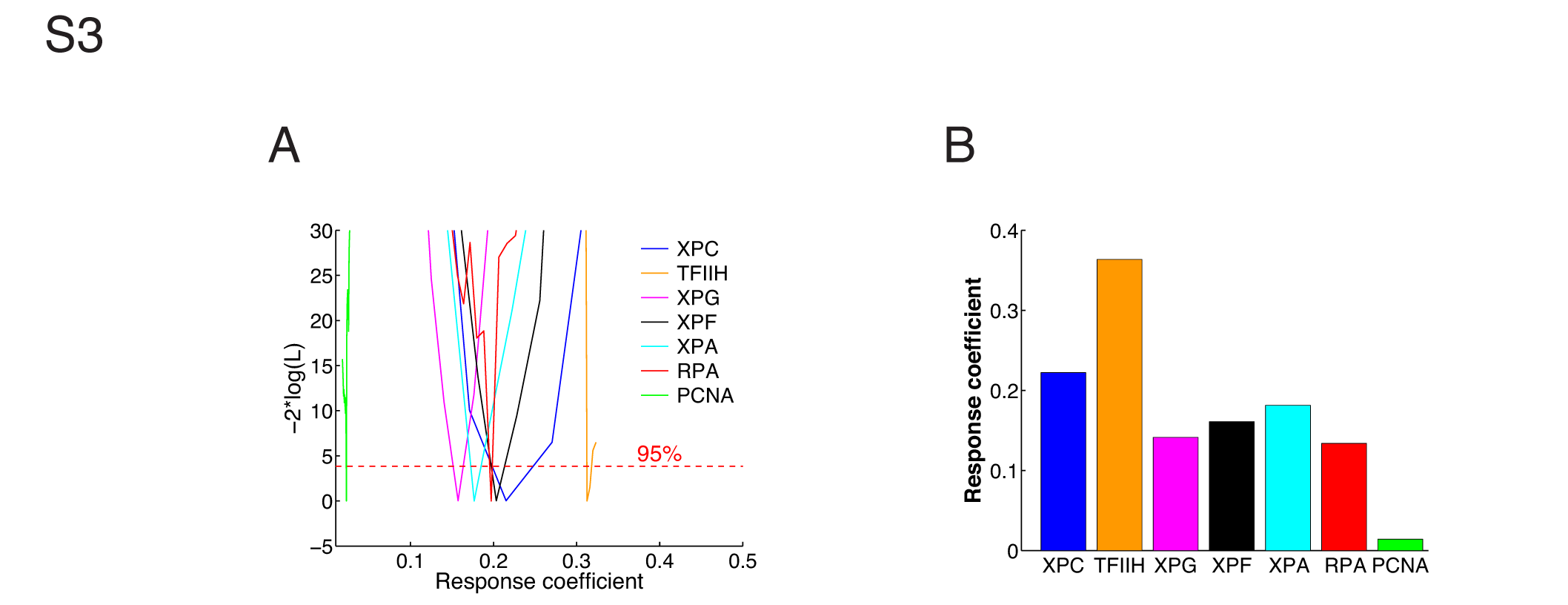

Supplement: Figure S3 — Repair-rate response coefficients are identifiable and show a similar distribution as incision-rate responds coefficients. (A) Prediction profile likelihoods and 95% confidence bounds for all 7 repair factors. (B) Response coefficients for the control of the incision rate by the concentrations of the repair factors. (TIF) [file pcbi.1003438.s003.tif]
