# Supplementary material for: Robustness of DNA Repair through Collective Rate Control
Source: PLoS Comput Biol. 2014 Jan 30;10(1):e1003438. doi: 10.1371/journal.pcbi.1003438 (PMC3907289; doi:10.1371/journal.pcbi.1003438)
Supplement: Table S1 — Values of binding and dissociation rate constants. NA, not applicable. kon, koff and KD (koff/kon) values are given for every repair protein and arranged in columns. Reference parameter set and 95% confidence intervals (in parentheses) are shown. Nuclear concentration (in micromolars) of NER factors are based on previously described data [8]. (PDF) [file pcbi.1003438.s004.pdf]

Table S1. **Values of binding and dissociation rate constants**

Value	XPC	TFIIH	XPG	XPF	XPA	RPA	PCNA
Concentration (μM)	0.140	0.360	0.440	1.110	0.170	1.110	1.110
**Damaged DNA**							
k_{on} ($\mu M^{-1} s^{-1}$)	0.025 (0.013;0.036)	0.2 (0.152;0.288)	NA	NA	NA	NA	NA
k_{off} (s^{-1})	0.231 (0.136;0.36)	0.01 (0.009;0.012)	NA	NA	NA	NA	NA
K_d (μM)	9.35 (3.46;16.09)	0.052 (0.044;0.072)	NA	NA	NA	NA	NA
**Unwound DNA**							
k_{on} ($\mu M^{-1} s^{-1}$)	0.022 (0.016;0.025)	0.049 (0.04;0.06)	0.037 (0.036;0.039)	0.0056 (0.0054;0.0059)	0.116 (0.109;0.125)	0.018 (0.016;0.02)	NA
k_{off} (s^{-1})	0.019 (0.019;0.019)	0.01 (0.009;0.012)	0.0146 (0.0140;0.0149)	0.0137 (0.0134; 0.0141)	0.017 (0.0167;0.0172)	0.022 (0.0213;0.0224)	NA
K_d (μM)	0.864 (0.635;1.006)	0.204 (0.163;0.259)	0.395 (0.373;0.419)	2.446 (2.344; 2.596)	0.147 (0.138;0.158)	1.222 (1.048;1.36)	NA
**Incised DNA**							
k_{on} ($\mu M^{-1} s^{-1}$)	0.022 (0.016;0.025)	0.049 (0.04;0.06)	0.037 (0.036;0.039)	0.0056 (0.0054;0.0059)	0.116 (0.109;0.125)	0.018 (0.016;0.02)	0.008 (0.0066;0.0111)
k_{off} (s^{-1})	0.019 (0.019;0.019)	0.01 (0.009;0.012)	0.0146 (0.0140;0.0149)	0.0137 (0.0134; 0.0141)	0.017 (0.0167;0.0172)	0.022 (0.0213;0.0224)	0.0031 (0.003;0.0032)
K_d (μM)	0.864 (0.635;1.006)	0.204 (0.163;0.259)	0.395 (0.373;0.419)	2.446 (2.344; 2.596)	0.147 (0.138;0.158)	1.222 (1.048;1.36)	0.388 (0.319;0.538)
**Resynthesized DNA**							
k_{on} ($\mu M^{-1} s^{-1}$)	NA	NA	NA	NA	0.022 (0.021;0.025)	0.032 (0.025;0.037)	0.004 (0.0038;0.005)
k_{off} (s^{-1})	NA	NA	NA	NA	0.0052 (0.0051;0.0053)	0.035 (0.031;0.042)	0.0026 (0.0025;0.0027)
K_d (μM)	NA	NA	NA	NA	0.236 (0.222;0.27)	1.167 (0.924;1.521)	0.605 (0.531;0.747)
**Rechromatinized DNA**							
k_{on} ($\mu M^{-1} s^{-1}$)	NA	NA	NA	NA	NA	0.065 (0.056;0.075)	0.396 (0.359;0.457)
k_{off} (s^{-1})	NA	NA	NA	NA	NA	0.035 (0.031;0.04)	0.061 (0.056;0.067)
K_d (μM)	NA	NA	NA	NA	NA	0.538 (0.438;0.645)	0.154 (0.134;0.182)
