# Supplementary material for: Robustness of DNA Repair through Collective Rate Control
Source: PLoS Comput Biol. 2014 Jan 30;10(1):e1003438. doi: 10.1371/journal.pcbi.1003438 (PMC3907289; doi:10.1371/journal.pcbi.1003438)
Supplement: Table S2 — Values of the enzymatic rate constants. Reference parameter set and 95% confidence intervals (in parentheses) are shown. In case of practical non-identifiability only the lower confidence bound is given. (PDF) [file pcbi.1003438.s005.pdf]

Table S2. **Values of the enzymatic rate constants**

| **Enzymatic rate** | | **$k_{cat}$** |
|---|---|---|
| | | $s^{-1}$ |
| Unwinding $\alpha$ | 19.9 | (>0.2) |
| Resynthesis $\gamma$ | 25.5 | (>1.5) |
| Rechromatinization $\delta$ | 0.001 | (0.001;0.0011) |
| Reannealing $\epsilon$ | 5.3 | (>0.9) |
