# Supplementary material for: Robustness of DNA Repair through Collective Rate Control
Source: PLoS Comput Biol. 2014 Jan 30;10(1):e1003438. doi: 10.1371/journal.pcbi.1003438 (PMC3907289; doi:10.1371/journal.pcbi.1003438)
Supplement: Text S1 — Mathematical modeling and analysis. (PDF) [file pcbi.1003438.s006.pdf]

Supplementary Text S1

1 Analytical model of DNA repair

We consider the simplified model of DNA repair depicted in Fig. 3: N proteins assemble to form a repair complex which then executes the repair reaction with rate constant ρ. Assembly of the individual components can be in a particular order (sequential) or without a specific order (random) or by a mixed mechanism with random and sequential subsystems. We consider completely sequential and completely random mechanisms as the extreme cases; Fig. 3 illustrates these two extremes for the case $N = 3$ (with the components of the repair complex A, B and C). Note that similar 'recruitment-reaction' schemes apply to other chromatin-associated processes including transcription initiation.

Sequential assembly proceeds via a single pathway compared to the large number of pathways for random assembly, which amount to $N!$ different N-step pathways for N protein components. For this reason, random assembly has an intrinsic advantage with respect to assembly speed. However, the sequential scheme has fewer incomplete protein complexes ($N-1$) than the random scheme ($2^N - 1$), thus favouring the complete, enzymatically active complex. The efficiency of random and sequential schemes in assembling the complete repair complex and carrying out the repair reaction will be affected by both these structural properties.

A second level of control is provided by the kinetic properties of the individual steps, measured by the on-rates of the proteins, their off-rates as well as the catalytic rate constant (ρ). Experimental measurements suggest that the apparent first-order on-rate constants (on-rate constants × free concentration of the component) are of the same order of magnitude as the off-rate constants (∼ 1/min; balanced reversibility of assembly), while catalysis of the reaction will be faster (∼ 1/s) [1]. We will contrast this realistic case with the hypothetical scenario that all proteins bind irreversibly.

To model the dynamics of repair, denote the composition of a macromolecular (multi-protein) complex in the assembly pathway by an index vector p, where a component $p_i = 1$ ($p_i = 0$) if protein i is present (absent) in the complex. For example, $p = (0, 1, 1, 0, \ldots)$ would denote a complex in which molecular component 1 and 4 are absent and components 2 and 3 are present. Furthermore, let $p^{(i)}$ denote a 'neighboring' index vector that differs from p only in position i (Hamming distance 1). The concentration of the complex with composition p, y_p is determined by the balance equation

$$\frac{dx_p}{dt} = \underbrace{\sum_{i(p_i=1)} \kappa_{p,i}\, c_i\, x_{p^{(i)}} - l_{p,i}\, x_p}_{\text{complex } p \text{ forms by binding of protein } i} \quad - \underbrace{\sum_{i(p_i=0)} \kappa_{p^{(i)},i}\, c_i\, x_p - l_{p^{(i)},i}\, x_{p^{(i)}}}_{\text{complex } p \text{ forms by dissociation of protein } i} \tag{1}$$

where the κ and l denote the association and dissociation rate constants, with units $M^{-1}s^{-1}$ and s^{-1}, respectively, and c_i is the concentration of unbound component i. For the fully assembled complex ($p = (1, 1, 1, \ldots)$), whose concentration we denote by $\hat{x}(t)$, the additional term $-\rho\hat{x}$ appears on the right-hand side of equation (1). For the concentrations of unbound components, we have

$$\frac{dc_i}{dt} = \sum_{i(p_i=1)} -\kappa_{p,i}\, c_i\, x_{p^{(i)}} - l_{p,i}\, x_p \tag{2}$$

The rate of the reaction catalysed by the multiprotein complex is given by $v(t) = \rho\,\hat{x}(t)$. The

mean time at which the reaction occurs can be defined by

$$\tau_i = \frac{\mu_1}{\mu_0} \tag{3}$$

with

$$\mu_k = \int_0^\infty t^k \, v(t) \, dt \tag{4}$$

denoting the kth moment of the reaction rate.

The mean reaction time τ can be calculated analytically when only a small fraction of any protein is bound to DNA at any time (i.e., $c_i(t) \approx$ constant), and all binding and dissociation rate constants are assumed equal ($\kappa_i c_i = k$ and $l_i = l$). Then integrating Eq. 1 to find the required zeroth and first moments of the catalytic rate yields linear systems of algebraic equations. We have solved them explicitly for $N \leq 20$; the solutions can be expressed as

$$\tau = \underbrace{\frac{1}{k}\sum_{i=0}^{N-1} A_i \left(\frac{l}{k}\right)^i}_{\text{first assembly}} + \underbrace{\frac{1}{\rho}\sum_{i=0}^{N} B_i \left(\frac{l}{k}\right)^i}_{\text{reassembly and reaction}} \tag{5}$$

for a complex of N proteins. When catalysis is fast compared to protein binding and dissociation ($\rho \gg k, l$), the reaction is limited by the rate of complex assembly, and only the first term is significant on the right-hand side in Eq. (5). This term, with coefficients A_i, gives the average time until the protein complex is fully assembled for the first time. The second term, with coefficients B_i, gives the additional time needed to carry out the reaction. It takes into account the possibility that the complete complex disassembles before the reaction takes place and must reassemble. The coefficients A_i and B_i depend on the assembly mechanism. We have for random and sequential assembly

$$A_i^{\text{rand}} = \sum_{j=1}^{N-i} \frac{1}{i+j} \frac{\binom{N}{j-1}}{\binom{N}{i+j}}, \quad B_i^{\text{rand}} = \binom{N}{i} \quad \text{and} \quad A_i^{\text{seq}} = N - i, \quad B_i^{\text{seq}} = 1, \tag{6}$$

respectively.

These analytical expressions provide insight into the dependence of the reaction time on the complex assembly mechanism and its parameters. In the limit of irreversible component binding, the sequential assembly time grows linearly with N while the random assembly time increases only logarithmically,

$$\tau_{\text{seq}}(l=0) = \frac{N}{k} + \frac{1}{\rho}, \quad \tau_{\text{rand}}(l=0) \approx \frac{1}{k}(0.577 + \ln N) + \frac{1}{\rho} \tag{7}$$

(Figure 3A). Thus irreversible assembly is always faster with a random mechanism, which is due to the larger number of potential assembly pathways. The larger number of incompletely assembled complexes in the random scheme are not detrimental to assembly speed when the components bind irreversibly.

For reversible component binding, the reaction is slower because protein complexes may disassemble and reassemble before the reaction takes place. This effect can become particularly pronounced with a large number of components N. However, for sequential assembly

the reaction time increases only as an algebraic function of N up to the case of balanced irreversibility, $l = k$, where

$$\tau_{\text{seq}}(l = k) = \frac{N(N+1)}{2k} + \frac{N}{\rho}. \tag{8}$$

For stronger reversibility $l > k$, the reaction time of the sequential mechanism grows exponentially with N.

For reversible binding of the proteins, random complex formation can still be faster than sequential assembly for a small number of components but eventually becomes much slower as the number of components grows (Figure 3B). In particular, for fast catalysis ($\rho \gg l, k$), random and sequential assembly mechanisms are of comparable efficiency for $N \leq 10$. Eqs (5) and (6) have been used to compute Fig. 3B; Eq. 1 with the simplifications $\kappa_i c_i = k$ ($c_i(t)$ = constant) and $l_i = l$ and $N = 9$ components has been used to compute Fig. 3C.

2 Data-based model of DNA repair

2.1 Model formulation

The model describes 5 DNA intermediates (I, damaged; II, unwound; III, incised; IV, resynthesized and V, rechromatinized). In total 7 repair proteins can bind to one or more repair intermediates (see Figure 4A). From now on we will use the following notation: XPC = C, TFIIH = T, XPG = G, XPF = F, XPA = A, RPA = R and PCNA = P. Apart from the restriction that TFIIH can bind only after lesions were detected by XPC [2] protein binding is assumed random and characterized by a protein binding constants k_i and a dissociation constants l_i. Transitions between different repair intermediates are described by catalytic rate constants (α, unwinding; ϵ, re-annealing; β, dual incision; γ, resynthesis; δ rechromatinization). We have the following system of equations for the protein complexes at the different repair intermediates (see also Luijsterburg et al. [1]):

$$\frac{d}{dt}\, y_\pi^R = \sum_p (-1)^{\pi(p)}\, l_\pi^R\, y_\pi^R\,|_{\pi(p)=1} + (-1)^{1+\pi(p)}\, k_\pi^R\, C_p(t)\, y_\pi^R\,|_{\pi(p)=0} + E(y_\pi^R) \tag{9}$$

The y_π^R variables denote the concentrations of the repair intermediate R to which the proteins described by index vector π are bound (see Section 1). We define $p \in \{C, T, G, F, A, R, P\}$ and have $\pi(p) = 1$ if the corresponding repair factor is bound and $\pi(p) = 0$ otherwise. The protein binding kinetics are governed additionally by the unbound nuclear concentrations C_p (analogous to Eq. 2). The enzymatic reaction rate $E(y)$ from one repair intermediate to the next is catalysed by a specific complex of repair proteins. If a state has no in- or outgoing enzymatic reactions then $E = 0$. For the transition from damaged to unwound DNA, we have

$$E(y_{00}^I) = \epsilon\, y_{0000000}^{II}, \quad E(y_{11}^I) = -\alpha\, y_{11}^I \tag{10}$$

For simplicity, we assume that the rate of incision is very fast and hence that as soon as all necessary enzymes are bound to the unwound state the lesion strand is rapidly incised ($\beta \to \infty$). This has the following consequences for unwound and incised DNA ($R = \text{II}$ and $R =$

III):

$$
\begin{aligned}
E(y^{II}_{110000}) &= \alpha\, y^{I}_{11} && (11)\\
E(y^{II}_{000000}) &= -\epsilon\, y^{II}_{000000} && (12)\\
\frac{d}{dt}\, y^{II}_{\pi(011111)} &= 0 && (13)\\
\frac{d}{dt}\, y^{III}_{\pi(0111110)} &= \sum_p (-1)^{\pi(p)}\, l^{III}_{\pi}\, y^{III}_{\pi}\,|_{\pi(p)=1} && (14)\\
&\quad + (-1)^{1+\pi(p)}\, k^{III}_{\pi}\, C_p(t)\, y^{III}_{\pi}\,|_{\pi(p)=0}\\
&\quad + \sum_p (-1)^{1+\pi(p)}\, k^{II}_{\pi}\, C_p(t)\, y^{II}_{\pi}\,|_{\pi(p)=0}\\
E(y^{III}_{0000111}) &= -\gamma\, y^{III}_{0000111} && (15)
\end{aligned}
$$

We further have for resynthesized DNA ($R = \text{IV}$):

$$E(y^{IV}_{111}) = \gamma\, y^{III}_{0000111}, \quad E(y^{IV}_{011}) = -\delta\, y^{IV}_{011}, \tag{16}$$

and for rechromatinized DNA ($R = \text{V}$):

$$E(y^{V}_{11}) = \delta\, y^{IV}_{011}. \tag{17}$$

The free nuclear protein concentrations are governed by 7 additional differential equations:

$$\frac{d}{dt} C_p = r \left(\sum_{R=I}^{V} \sum_{\pi} \delta_{p1}\, l^{R}_{\pi}\, y^{R}_{\pi} - \delta_{p0}\, k^{R}_{p}\, C_p\, y^{R}_{\pi} \right), \tag{18}$$

where we have used the Kronecker δ to ensure binding of the proteins to the correct intermediate. The factor r takes account of the volume ratio of local damage to nuclear volume (estimated to be about 0.1).

In total the model comprises 206 distinct DNA repair states and has 31 binding and dissociation parameters. As initial conditions we used the nuclear concentrations shown in Table SI and an average concentration of 3.33 μM for the amount of inflicted damages [1]. To quantify the accumulation of a particular repair protein we summed up the states where the protein is bound. For the representation of the FLIP experiments we assumed that each protein dissociating from the repair intermediate is rapidly bleached. Thus, in the model representation all k^{R}_{i} for the respective protein were set to zero from the time when bleaching was started. The starting point of the FLIP experiment depends on the time when the repair protein reached maximal accumulation (600s for XPC and ERCC1-XPF, 900s for XPG and TFIIH, 2,000s for XPA, and 7,200s for RPA and PCNA).

2.2 Parameter fitting and identifiability analysis

We performed the identifiability analysis by calculating the profile likelihood estimate (PLE) for all 31 parameters. Detailed description how to implement and perform PLE can be found in Raue et al. [3]. Initial fitting was performed by maximizing the likelihood with the MATLAB implementation of the trust-region method and user-supplied derivatives [4]. To reach a global minimum we began the fitting procedure from distant locations in parameter space by Latin-Hypercube sampling of the initial parameter values. Starting from the best fit for each PLE

the current parameter was fixed stepwise in ascending and descending direction. At every step all other parameters were locally re-fitted simultaneously. A parameter was determined as identifiable if the likelihood profile crossed the 95 % confidence level drawn from a χ^2 distribution (all binding and dissociation rate constants of the proteins). For the enzymatic rate constants, only a lower bound was found, implying that these parameters need to be sufficiently fast. This case of practical non-identifiability proved without consequence for the goodness of the predictions made with the model (Section 2.3).

2.3 Prediction profile likelihoods

To determine the confidence bounds for the responds coefficients (RC) we calculated their Prediction Profile Likelihood (PPL). To this end, we fixed the value for the RC prediction. This value is increased or decreased stepwise, and this nonlinear constraint is used as an additional penalty during the least square fitting procedure. For each step all remaining model parameters are fitted simultaneously until the convergence is reached. A detailed description about implementation of this method can be found in the supplemental material of Kreutz et al. [5].

3 Estimating the measurement error for fluorescence-microscopy quantification of repair factors

To dissect the natural variability of XPC from the measurement error we correlated the GFP-tagged expression values (I_x) with the immunofluorescence signal (I_y). We assume that both quantities have a normalized error $x_{\mathrm{err}} \sim y_{\mathrm{err}} \rightarrow \mathrm{Normal}(0, p(I_{x,y}))$ that can be decomposed into measurement error and natural variability. Measuring GFP and immunofluorescence signal independently at the same time, the measurement error should be orthogonal to the natural variability, which is equal in both measurements. Thus, we approximate the measurement error geometrically by

$$p_{\mathrm{merr}} = \sigma\left(\frac{1}{\sqrt{2}}\left(\frac{I_x}{I_E} - \frac{I_y}{I_E}\right)\right), \quad \text{where } I_E = \frac{1}{2}(I_x + I_y). \tag{19}$$

4 Derivation of Equation [4]

Consider the repair rate v as a function of the concentration of the repair factors C_i and the initial amount of DNA lesions L:

$$v = Lf(C_1, \ldots, C_N) \tag{20}$$

The dependence on L is linear as we have shown here that the repair is first-order in the amount of inflicted lesions. Let us assume that L and C_i vary independently between different cells. According to the standard law for propagation of uncertainty, the resulting variability in v is approximated by

$$\sigma(v) = \sqrt{\sum_i \left(\frac{\partial v}{\partial C_i}\sigma(C_i)\right)^2 + \left(\frac{\partial v}{\partial L}\sigma(L)\right)^2}, \tag{21}$$

where $\sigma(x)$ denotes the standard deviation of x. Introducing the response coefficients

$$R_i = \frac{C_i}{v}\frac{\partial v}{\partial C_i}$$

and noting that $\sigma(v)/v = CV(v)$ and $\sigma(C_i)/C_i = CV(C_i)$ are the coefficients of variation, Eq. 21 can be rewritten in the form

$$CV(v) = \sqrt{\sum_i (R_i CV(C_i))^2 + CV(L)^2} \tag{22}$$

Obviously, the 'response coefficient' for the initial amount of inflicted lesions is 1.

References

[1] Luijsterburg MS, et al. (2010) Stochastic and reversible assembly of a multiprotein DNA repair complex ensures accurate target site recognition and efficient repair. J Cell Biol 189: 445–463.

[2] Nishi R, et al. (2009) UV-DDB-dependent regulation of nucleotide excision repair kinetics in living cells. DNA Repair 8: 767–776.

[3] Raue A, et al. (2009) Structural and practical identifiability analysis of partially observed dynamical models by exploiting the profile likelihood. Bioinformatics 25: 1923–1929.

[4] Coleman TF, Li Y (1996) An interior trust region approach for nonlinear minimization subject to bounds. SIAM J Optim 6: 418–445.

[5] Kreutz C, Raue A, Timmer J (2012) Likelihood based observability analysis and confidence intervals for predictions of dynamic models. BMC Syst Biol 6: 120.
